# Interferon β-1a for the treatment of Ebola virus disease: A historically controlled, single-arm proof-of-concept trial

**DOI:** 10.1371/journal.pone.0169255

**Published:** 2017-02-22

**Authors:** Mandy Kader Konde, Darren P. Baker, Fode Amara Traore, Mamadou Saliou Sow, Alioune Camara, Alpha Amadou Barry, Doussou Mara, Abdoulaye Barry, Moussa 3 Cone, Ibrahima Kaba, Amento Ablam Richard, Abdoul Habib Beavogui, Stephan Günther, on behalf of European Mobile Laboratory Consortium, Melania Pintilie, Eleanor N. Fish

**Affiliations:** 1 Sustainable Health Foundation (FOSAD), Conakry, Guinea; 2 Center of Excellence for Training on Research and Priority Diseases (CEFORPAG), Conakry, Guinea; 3 Sanofi Genzyme, Cambridge, Massachusetts, United Staes of America; 4 Infectious Disease Ward, National Donka Hospital, Conakry, Guinea; 5 Bernhard-Nocht-Institute for Tropical Medicine, Hamburg, Germany; 6 Department of Biostatistics, University Health Network, Toronto, Canada; 7 Toronto General Research Institute, University Health Network, Toronto, Canada; 8 Department of Immunology, University of Toronto, Toronto, Canada; Azienda Ospedaliera Universitaria di Perugia, ITALY

## Abstract

To date there are no approved antiviral drugs for the treatment of Ebola virus disease (EVD). Based on our *in vitro* evidence of antiviral activity of interferon (IFN)-ß activity against Ebola virus, we conducted a single arm clinical study in Guinea to evaluate the safety and therapeutic efficacy of IFN β-1a treatment for EVD. Nine individuals infected with Ebola virus were treated with IFN β-1a and compared retrospectively with a matched cohort of 21 infected patients receiving standardized supportive care only during the same time period at the same treatment unit. Cognizant of the limitations of having treated only 9 individuals with EVD, the data collected are cautiously considered. When compared to supportive care only, IFN β-1a treatment seemed to facilitate viral clearance from the blood and appeared associated with earlier resolution of disease symptoms. Survival, calculated from the date of consent for those in the trial and date of admission from those in the control cohort, to the date of death, was 19% for those receiving supportive care only, compared to 67% for those receiving supportive care plus IFN β-1a. Given the differences in baseline blood viremia between the control cohort and the IFN-treated cohort, an additional 17 controls were included for a subset analysis, from other treatment units in Guinea, matched with the IFN-treated patients based on age and baseline blood viremia. Subset analyses using this expanded control cohort suggests that patients without IFN β-1a treatment were ~ 1.5–1.9 fold more likely to die than those treated. Viewed altogether the results suggest a rationale for further clinical evaluation of IFN β-1a.

## Introduction

Zaire Ebola virus (EBOV), a member of the filovirus family, causes severe, frequently lethal infections in humans and primates [1]. Since December 2013 the outbreak of Ebola virus disease (EVD) in West Africa has claimed 11,314 lives of the 28,630 confirmed cases. Clinical symptoms at onset of EVD include headache, fever, asthenia, arthralgia and myalgia. Gastrointestinal symptoms including abdominal pain, nausea, vomiting and diarrhea develop, leading to an electrolyte imbalance associated with intravascular volume depletion. Indeed, EVD is associated with profound endothelial dysfunction leading to fluid shifts that may result in cardiovascular collapse and renal failure [2,3]. Notably, the overall fatality rates differed in the 3 countries with the greatest number of confirmed cases: 67% in Guinea, 45% in Liberia and 28% in Sierra Leone [4]. Variables such as extent of viremia, time to onset of supportive care, and the level of supportive care, contributed to the case fatality ratio [5]. In the absence of any approved treatments, the high case fatality ratio prompted the consideration of potential treatment options, including the repurposing of approved drugs.

The type I interferons (IFNs) IFN-α and IFN-β, exhibit broad spectrum antiviral activity, with demonstrated clinical effectiveness against HBV, HCV, influenza A viruses and the SARS-CoV [6]. IFN-α/β production occurs as the earliest non-specific response to viral infection, directly inhibiting viral infection and activating the innate and adaptive immune responses to clear virus. Indeed, viruses have evolved immune evasion strategies specifically targeted against the type I IFN response, confirming the importance of IFNs as antivirals. This immune evasion strategy is particularly relevant when one considers IFN and EBOV infection. Experimental data indicate that the EBOV proteins VP24 and VP35 inhibit host cell systems that lead to type I IFN production and also inhibit events associated with an IFN response [7–9]. Accordingly, early post-exposure treatment with type I IFN might override these inhibitory effects of EBOV. IFN β-1a therapy has been shown to prolong survival in a rhesus macaque model of lethal EVD [10] and IFN-α2b treatment reduced viremia and extended the time to death in a similar cynomolgus model of lethal EVD [11]. A combination of monoclonal antibodies targeted against EBOV (ZMAb) protects macaques from lethal EVD, with decreasing efficacy if treatment is delayed to 2 days post-infection [12]. Addition of an adenovirus expressed vectored IFN-α to ZMAb extended the treatment window and improved protection [12]. Moreover, in mice and guinea pigs, using this replication-deficient human adenovirus expressing recombinant IFN-α alone, post-exposure treatment elicited full protection from lethal doses of the mouse- and guinea pig- adapted EBOV [13]. Notably, examination of serum samples from infected individuals during the 2000 outbreak of Sudan EBOV in Uganda revealed that surviving patients had significantly higher levels of IFN-α within the first few days of the onset of critical illness [14].

Viewed altogether, these studies prompted our evaluation of IFN treatment for EVD. In studies using transcription competent virus-like particles and infectious eGFP-Ebola virus, we provided evidence that IFN-α/ß limit Ebola virus (EBOV) infection *in vitro*, and showed that IFN ß-1a exhibits superior antiviral potency compared with IFN-α [15]. These findings provided the basis for this single arm proof-of-concept pilot study to evaluate the safety and efficacy of IFN-β treatment for EVD. This study was undertaken in an ETU close to Coyah, Guinea, during the 2014–2015 Ebola outbreak. While a placebo-controlled study was not permitted by the Guinean Health Authorities due to ethical concerns, laboratory and clinical data from EBOV-infected individuals who received only supportive care at the same treatment center and during the same period as those who received IFN β-1a was available for comparison. We provide preliminary evidence supporting further evaluation of IFN β-1a in any subsequent outbreak.

## Materials and methods

### Study design

Given (i) the urgent need to introduce drugs that would reduce mortality of EVD, (ii) the limited data on the clinical and biochemical parameters associated with EVD and (iii) the limited care provisions available in the treatment units during the outbreak, the study design focused on the objective endpoints of reduction in blood viremia, resolution of clinical symptoms, improvement in survival and safety of IFN β-1a treatment.

### Study protocol & informed consent

The study protocol (English version;) is provided as Supplementary Materials. Approvals were obtained from the Guinean Ministry of Health (#0777/CNRE; Dr. Sakoba Keita) (February 29, 2015), the CNERS, Guinea (016/CNERS/15; Prof. Oumou Younoussa Sow) (February 16,2015) and the Ebola Research Commission, National Public Health Institute, Guinea (Dr. Lamine Koivogui) (December 12, 2014). Written informed consent was obtained from all patients who received IFN β-1a treatment.

### Trial registration

ISRCTN 17414946. This trial registration was delayed. The Guinean Health Ministry registered the trial as #0777/CNRE on February 29, 2015.

### Patient selection

The initial study population involved patients who were admitted to the Ebola Treatment Unit (ETU) in a rural area close to the town of Coyah, in west Guinea, during the period March 26, 2015 –June 12, 2015. Inclusion (eligibility) criteria for IFN β-1a therapy were (1) symptom onset within 6 days, (2) Blood RT-PCR-confirmed positive for EBOV, (3) patient/designate informed consent for use of IFN β-1a. Exclusion criteria included (1) symptom onset more than 6 days prior to admission, (2) age < 17 or >70 years, (3) contra-indication to use of IFN β-1a or any of the constituents of the drug product. Recruitment of patients was difficult in the declining outbreak, limiting this proof-of-concept study to a pilot study. Nine patients met the inclusion criteria for IFN β-1a treatment. A cohort of historical controls of 28 patients was available for comparison with the treated patients. 7 patients of this cohort were excluded: 4 were younger than 17 years of age, 2 had onset of symptoms more than 6 days before admission and 1 was older than 70 years of age. The 21 control patients were admitted to the Coyah ETU during the same time period as the IFN β-1a treated patients, with RT-PCR-confirmed blood EBOV (Table 1). We also included an additional 17 patients who matched the IFN-treated patients for eligibility criteria based on ≤ 6 days from symptom onset, age, and under care in a Guinean treatment centre, who were better matched for baseline CT values. Since no data were available to us on serial CT values for these additional patients, vital status only (alive or dead) was used as the outcome.

**Table 1:** Patient characteristics.

Variable	Type of summary	Control cohort n = 21	IFN β-1a treated cohort n = 9	p-value
Age (years)	median (range)	35 (20–70)	38 (18–50)	0.41
Sex	Female	14 (66.7%)	5 (55.6%)	0.69
	Male	7 (33.3%)	4 (44.4%)	
Days from symptom onset	median	2 (0–5)	2 (0–6)*	0.85
CT 0	Median (range)	17.9 (14–26.5)	22.1 (16.2–30.6)	0.012

*one patient was asymptomatic at time of PCR confirmation of EVD

### Treatment protocol

Eligible patients were administered IFN β-1a (0.5 mL liquid formulated AVONEX drug product, Biogen) subcutaneously daily. Refer to Table 2 for dosing schedules. Each 0.5mL contained 30μg (6 x 10^6^ IU) of IFN β-1a. All patients treated with IFN β-1a and all historic controls received supportive care as was available in this resource constrained setting, as needs demanded, comprising: rehydration solution (oral), Ringer’s lactate solution (perfused), isotonic salt /glucose solutions (perfused), pain and fever medication: Novalgin (iv), Paracetamol (oral), Antalgin (oral) orTramadol (oral), Plumpy’Nut therapeutic diet (oral), vitamin B complex (perfused), vitamin C (iv / perfused), Cimetidine (iv), Omeprazol (oral) or iv metoclopramide (to treat symptoms of gastroesophageal reflux), Vogalene (iv) (to treat symptom of nausea and vomiting), Cimetidine (iv) (inhibits stomach acid production), Dycinone (iv) (an antihemorrhagic), cephalosporin antibiotics Cefixime (oral) and Ceftriaxone (iv), antibiotic Metronidazole (perfused) and the anti-malarial Coartem (oral) (Refer to S1 Excel File for details of supportive care provided to IFN-treated patients). Notably, all historic controls received similar supportive care as IFN-treated patients. Pain and fever were managed on a case by case basis, as were gastrointestinal issues, nutritional requirements, co-infections and hemorrhagic occurrences. Of importance is that each patient that we report on, whether a participant in the IFN treatment clinical study, or a historic control, received supportive care as outlined.

**Table 2:** Characteristics of IFN β-1a treated patients.

Patient	Age (years)	Sex	Days from symptom onset to 1^st^ dose	Number of IFN doses		Total μg	CT0 value prior to 1^st^ dose	Days from CT0 to 1^st^ dose	Days from 1^st^ IFN dose to 1st PCR negative	Outcome
				30μg	15μg					
IFN 01	50	Female	3	3	-	90	26.78	2	n/a	deceased
IFN 02	18	Male	6	10	-	300	22.09	1	9	alive
IFN 03	50	Female	4	2	-	60	16.17	1	n/a	deceased
IFN 04	40	Female	3	3	2	120	30.61	1	2	alive
IFN 05	38	Female	3	8	2	270	28.11	1	6	alive
IFN 06	50	Female	3	10	-	300	24.13	1	9	alive
IFN 07	20	Male	1*	9	-	270	20.10	1	n/a	deceased
IFN 08	18	Male	5	17	-	510	21.86	1	14	alive
IFN 09	21	Male	3	13	-	390	21.25	1	10	alive

* patient asymptomatic when CT0 determined

Patients were discharged on resolution of clinical symptoms and following 2 consecutive negative blood RT-PCR results for viremia (≥ CT value of 40), 48 hours apart, as per WHO guidelines [16].

### Adverse events, reporting and management

Please refer to the Study S1 Protocol. Briefly, adverse events as have been reported are described in the product monograph for AVONEX. The study protocol indicated dose adjustment or withdrawl for severe adverse events (worsening and severe clinical symptoms associated with EVD (including persistently elevated AST and ALT) and/or resolution of disease (CT value ≥ 40).

### Laboratory studies

Laboratory investigations included serial blood RT-PCR analyses for EBOV and biochemical assays. Blood draws into EDTA tubes were immediately processed for determination of viremia. EVD diagnosis was made using a semi-quantitative RT-PCR assay (RealStar Filovirus Screen RT-PCR kit 1.0, altona Diagnostics, GmbH). Measurements are expressed as CT values (cycle threshold), inversely proportional to viral load. The CT value for ebola virus positivity was ≤ 40.

### Statistical analysis

Baseline characteristics and treatment of the control and IFN β-1a treated groups are presented as medians, ranges or numbers and percentages for all clinical parameters, biochemistry values and symptoms. The comparison between the two cohorts was performed utilizing either the Mann-Whitney test for the continuous variables (e.g. CT values) or the Fischer exact test for the categorical variables. Survival percentages and plots were based on Kaplan-Meier estimates. The survival curves were compared using the log-rank test (p-value shown). The IFN β-1a treatment as well as the CT value (as continuous) were also tested using the Wald test within the Cox proportional hazards model. The effect of IFN β-1a treatment was tested adjusting the model for the CT value. The assumptions of proportionality of hazards and linearity (for CT value) were inspected and no departure was found. The rate of change over time for CT values and fever as well as the differences for these rates between the IFN β-1a treatment and the control cohort were tested utilizing the mixed effect modeling. This type of modeling can account for the possible correlations between observations belonging to the same individual. On inspection of the residuals no departure from normality was observed.

The difference in the change of symptoms over time between the treatment group and controls was investigated using general estimating equation (gee) models with the logit link (specific to binary outcome). The presence of a symptom was the outcome and the treatment, day and their interaction were the covariates. The interaction term measures the difference in the rate of change of the symptom occurrence between the two groups (controls vs IFN β-1a treated). The interaction p-values were adjusted for multiple comparisons utilizing Hochberg approach [17]. Analysis was performed using R-3.2.2. The packages needed for this analysis were gee and NIME.

## Results

**Fig 1:**
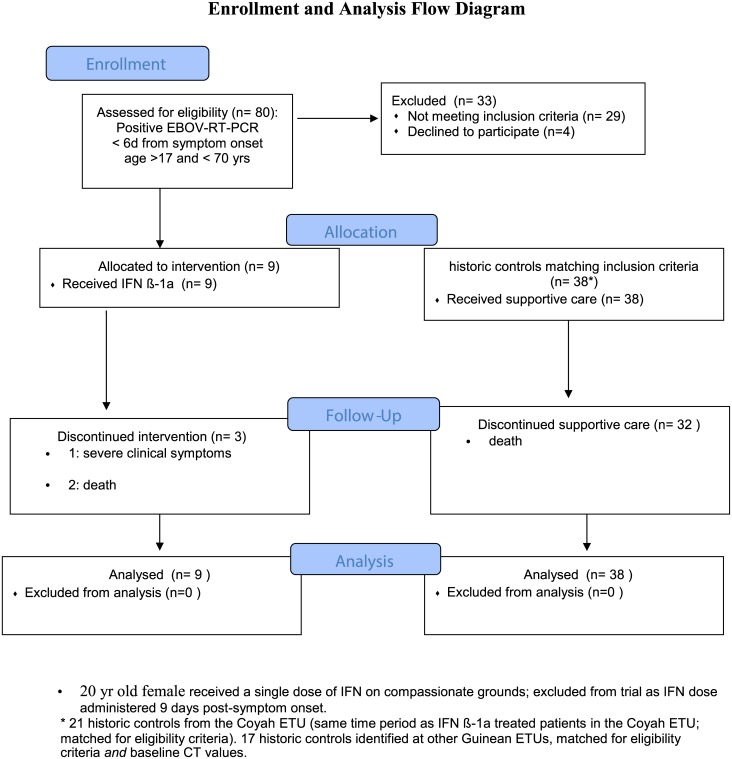
Enrollment and analysis flow diagram.

Refer to Fig 1. The initial study population included 19 females and 11 males, aged 18 to 70 years at the Coyah Treatment Unit, Guinea. EBOV was confirmed in all study participants using blood RT-PCR. It is important to note that while IFN β-1a was available as a treatment option, EBOV-infected individuals and/or their designate had the right to refuse IFN β-1a treatment, which 4 did. The IFN β-1a treatment and control groups did not differ in age, sex or day of admission relative to symptom onset (Table 1). Elevated alanine aminotransferase (ALT), aspartate aminotransferase (AST), creatinine (CRE) and C reactive protein (CRP) levels were consistent features of EVD in all study participants. Of the 9 patients treated with IFN β-1a, 5 started IFN treatment on day 3 post-symptom onset, 1 on day 4 post-symptom onset, one on day 5 post-symptom onset and one on day 6 post-symptom onset. One patient started treatment the next day following admission to the treatment unit based on being a high risk contact who was asymptomatic, but was PCR positive. Given the elevated ALT, AST and CRE levels in all patients with EVD, we were unable to determine whether IFN β-1a treatment negatively affected these biochemical blood measurements. 1 patient (Table 2, #4) with a PCR negative result received a dose reduction (3 x10^6^ IU), related to elevated AST and ALT levels, and had a second 2 consecutive PCR negative result on this reduced dose. Another patient (#5) received a dose reduction (3x10^6^ IU) because of rapid resolution of clinical symptoms and a coincident first negative PCR result. This patient also had a consecutive PCR negative result on the reduced IFN β-1a dose. 1 patient had treatment discontinued because of severe clinical symptoms and subsequently died. 2 patients received treatment beyond 10 days, until they became PCR negative (Table 2). 3 of the 9 patients who received IFN β-1a died. Notably, ALT, AST and CRE were significantly elevated in all 3 patients who died, despite one patient (#7) exhibiting reduction in blood viremia from a CT value of 20.1 at start of IFN β-1a treatment, to a CT value of 38 on day 10, when the patient succumbed to disease (Table 2).

**Fig 2:**
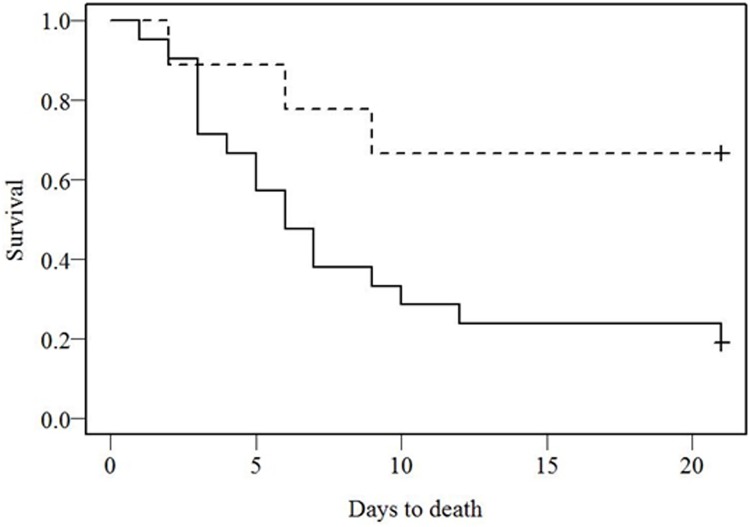
IFN β-1a treatment effect on survival of patients with EVD. Survival curves for patients with EVD receiving supportive care only (n = 21) (-----) or supportive care plus IFN β-1a treatment (n = 9) (-----). Survival was calculated from the date of consent for those receiving IFN β-1a treatment and date of admission for those in the control cohort, to the date of death. Survival plots were based on Kaplan-Meier estimates and the plots were compared using the log rank test (p = 0.026).

The data in Fig 2 show survival percentages for the control (19% at 21 days) and IFN β-1a treated patients (67% at 21 days, log-rank p = 0.026). The hazards ratio (HR) for the treatment when alone in the model is 0.27, 95%CI:0.08–0.94, p = 0.039 (Wald test). This analysis suggests that the treatment is beneficial to the patients with a HR of 0.27 (those untreated are approximately 3.7-times more likely to die). Age and sex did not affect survival. The HR for the CT values, as continuous, when alone in the model, for the untreated control cohort is 0.8, 95% CI:0.62–0.97, p = 0.0234. These results indicate that CT values have a prognostic effect, with larger values being associated with better survival. For each unit decrease of CT, the risk of death increases 1.25 fold. When the model is adjusted for CT values, the effect of the treatment becomes weaker (HR = 0.79) and for CT slightly stronger (HR = 0.76 for each unit of CT value increase). This analysis suggests that for the same CT values the risk of dying for the untreated is 1.26 fold compared to the IFN treated group, and each unit decrease in CT value will increase the risk of dying by 1.31 fold. For a CT value of 19 (median for CT) the probability of dying for those untreated was 1.35 fold larger than for those treated. Due to the smaller number of deaths in this cohort, it is not possible to adjust the model for more covariates.

Given that a recent analysis of baseline CT values supports our findings of their prognostic effect [18], we extended our analysis for treatment effect on survival as the outcome, using an expanded cohort of untreated and infected patients. We included an additional 17 patients who matched the IFN-treated patients for eligibility criteria based on ≤ 6 days from symptom onset, age, and under care in a Guinean treatment centre, who were better matched for baseline CT values. Notably, these patients were never approached to participate in the IFN β-1a treatment trial. Since no data were available to us on serial CT values for these additional patients, we only used vital status (alive or dead) as the outcome. Three logistic regression analyses were performed: (i) An analysis in which all patients were included: 9 IFN-treated and 38 controls. The results suggest that the treatment is significant even when the model is adjusted for CT value. The patients without treatment had an odds ratio of dying 7.54 fold more likely than those treated, when adjusting for the CT value. For a CT value of 20 (median for CT) the probability of dying for those untreated was 1.89 fold larger than for those treated (S1 Table) (ii) An analysis in which patients older than 50 years (which is the maximum age in the IFN-treated group) and with baseline CT values <16 or >31 (the range of values for the IFN-treated group) were excluded. The patients without treatment had an odds ratio of dying 6.39 fold more likely than those treated, when adjusting for the CT value. For a CT value of 20 (median for CT) the probability of dying for those untreated was 1.81 fold larger than for those treated (S2 Table). (iii) A matched analysis. Each patient from the IFN-treated group was matched to a group of patients in the control group such that the age difference was not larger than 5 years and the CT difference was not larger than 2. For 5 patients suitable matches were found: For IFN 02 one match, for IFN 03 one match, for IFN 04 three matches, for IFN 08 two matches and for IFN 10 three matches. For IFN 05, IFN 06, IFN 07 and IFN 09 no matches were found and they did not enter this analysis. The effect of the treatment based on the conditional logistic regression is significant (OR = 0.17, p = 0.012). The patients without treatment had an odds ratio of dying 6.0 fold more likely than those treated. The probability of dying for those untreated was 1.5 fold larger than for those treated (S3 Table).

**Fig 3:**
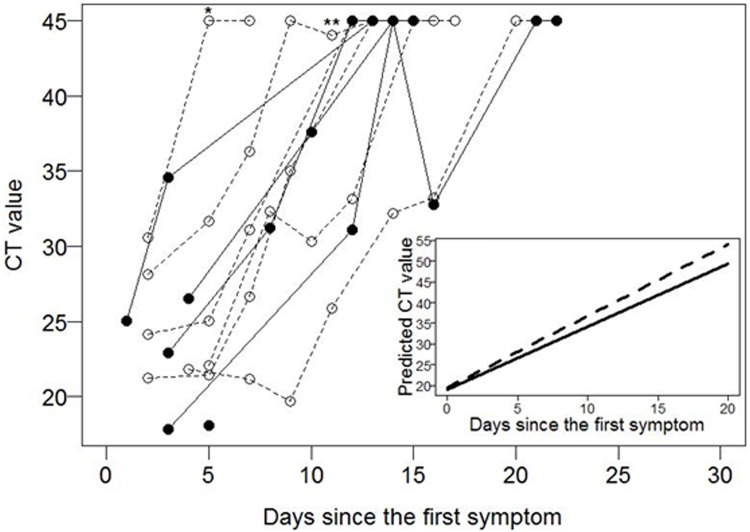
Effect of IFN β-1a treatment on viral clearance from the blood. Serial quantitative PCR CT (cycle threshold) values in venous blood samples from study participants that became PCR negative (survivors). * dose reduction due to elevated AST and ALT levels; ** dose reduction due to rapid resolution of EVD; ___ controls; ------IFN β-1a treated. inset: Fitted plots for CT values for each group.

Next, the rate of viral clearance from blood was evaluated, using the original 21 control cohort, for whom we had serial data (Fig 3). To remove bias associated with CT values from patients who succumbed to disease, i.e. did not clear virus, this analysis was performed only on patients who were alive at the end of the study, i.e. cleared virus. We observe a trend of faster clearance in the IFN β-1a treated patients compared with the controls (Fig 3, inset). The CT values increase by 1.53 units/day in the controls and by 1.62 units/day for the IFN treated patients. The difference (0.19) is not statistically significant (p = 0.36).

Patient symptoms were recorded in the morning, afternoon and evening over time for all study participants. Refer to Table 3. Frequency and number of clinical symptoms are described in S4 Table. Overall, the data in Table 3 indicate that IFN β-1a treatment led to earlier resolution of many clinical symptoms, including those associated with the gastrointestinal dysfunction of EVD, namely abdominal pain, vomiting and diarrhea. In the context of fever, temperature values fluctuated between 34° and 40.5° among all study participants (data not shown). Fever did not decrease differently between the two groups, with the interaction p-value s = 0.069, 0.63, 0.82, for the morning, afternoon and evening, respectively. Overall, in both groups fever seemed to decrease by a small amount in the afternoon (0.07°C, p<0.001). No statistically significant decrease was observed in the two groups for the temperatures taken in the morning or evening (p = 0.069 and p = 0.93, respectively). Pulse taken in the afternoon decreased by 0.6 per day in the control cohort (p = 0.19) and increased by 1.1 per day in the IFN β-1a treated group (p = 0.0042). The difference between these rates (interaction term) was statistically significant (p = 0.0053). The rate of change for the morning or evening pulse did not change significantly between the two groups over time (p = 0.33 and 0.19, respectively). Similarly, the rate of change for systolic blood pressure was not different between groups (p = 0.37 for morning, p = 0.92 for afternoon and p = 0.49 for the evening measure).

**Table 3:** Effects of IFN β-1a treatment on clinical symptoms of EVD.

Symptom	Control patients OR (95% CI, p-value) n = 21	IFN β-1a treated patients OR (95% CI, p-value) n = 9	Difference of effect (interaction p value)
Headache am	1.3(1.02–1.66,p = 0.034)	0.84(0.61–1.16,p = 0.29)	0.034
Headache pm	0.84(0.7–1,p = 0.044)	0.87(0.63–1.21,p = 0.41)	0.81
Headache evening	1.01(0.8–1.27,p = 0.96)	0.83(0.58–1.18,p = 0.29)	0.36
Asthenia am	1.13(0.92–1.4,p = 0.25)	0.68(0.53–0.87,p = 0.0027)	0.0023
Asthenia pm	1.07(0.9–1.27,p = 0.47)	0.65(0.54–0.77,p = 1.5e-06)	0.000076
Asthenia evening	0.98(0.84–1.14,p = 0.79)	0.68(0.53–0.87,p = 0.0021)	0.013
Muscle pain am	1.04(0.88–1.23,p = 0.66)	0.92(0.77–1.11,p = 0.4)	0.36
Muscle pain pm	0.92(0.79–1.07,p = 0.29)	0.94(0.83–1.07,p = 0.35)	0.81
Muscle pain evening	0.84(0.7–1,p = 0.056)	0.98(0.71–1.36,p = 0.92)	0.4
Anorexia am	1.12(0.94–1.33,p = 0.21)	0.65(0.46–0.92,p = 0.016)	0.0065
Anorexia pm	1.15(1.01–1.32,p = 0.038)	0.8(0.7–0.91,p = 0.001)	0.00016
Anorexia evening	1(0.86–1.17,p = 0.99)	0.83(0.64–1.09,p = 0.18)	0.25
Nausea am	1.01(0.73–1.39,p = 0.95)	0.63(0.6–0.67,p = 0)	0.0046
Nausea pm	0.81(0.66–1,p = 0.052)	0.52(0.5–0.54,p = 0)	0.000058
Vomiting am	1.04(0.88–1.23,p = 0.64)	0.63(0.5–0.79,p = 6.1e-05)	0.00042
Vomiting pm	0.82(0.7–0.95,p = 0.007)	0.57(0.43–0.77,p = 2e-04)	0.035
Vomiting evening	0.9(0.77–1.06,p = 0.22)	0.81(0.63–1.05,p = 0.12)	0.49
Diarrhea am	1.19(0.99–1.44,p = 0.067)	0.59(0.45–0.77,p = 8e-05)	0.00002
Diarrhea pm	1.01(0.86–1.2,p = 0.87)	0.6(0.5–0.74,p = 5e-07)	0.000085
Diarrhea evening	1.05(0.88–1.26,p = 0.57)	0.57(0.49–0.66,p = 3e-13)	0.00000025
Dyspnea pm	1.04(0.78–1.39,p = 0.77)	1.35(0.87–2.1,p = 0.18)	0.34
Dyspnea evening	1.05(0.85–1.29,p = 0.67)	1.42(0.98–2.07,p = 0.064)	0.16
Cough am	1.29(1.03–1.61,p = 0.027)	0.88(0.79–0.99,p = 0.031)	0.0033
Cough pm	1.21(0.87–1.69,p = 0.25)	0.91(0.75–1.11,p = 0.36)	0.14
Cough evening	1.28(0.97–1.7,p = 0.081)	0.99(0.82–1.19,p = 0.9)	0.13
Thoracic pain am	0.8(0.75–0.85,p = 1.8e-11)	0.89(0.71–1.11,p = 0.31)	0.36
Thoracic pain pm	0.55(0.32–0.95,p = 0.034)	0.95(0.73–1.25,p = 0.74)	0.079
Vertigo evening	0.8(0.35–1.83,p = 0.59)	0.66(0.42–1.05,p = 0.08)	0.71
Abdominal pain am	1.13(1–1.28,p = 0.055)	0.49(0.41–0.59,p = 3.7e-15)	5.2E-14
Abdominal pain pm	0.84(0.75–0.95,p = 0.0036)	0.55(0.43–0.69,p = 7.8e-07)	0.0013
Abdominal pain evening	0.99(0.86–1.14,p = 0.88)	0.47(0.32–0.7,p = 0.00015)	0.00046
Dehydration evening	1.08(0.95–1.23,p = 0.25)	0.52(0.5–0.54,p = 0)	0
Hemorrage am	1.08(0.88–1.33,p = 0.44)	1.24(0.97–1.6,p = 0.089)	0.41
Hemorrage pm	0.95(0.8–1.14,p = 0.61)	1.25(0.99–1.57,p = 0.057)	0.071
Hemorrage evening	0.99(0.72–1.36,p = 0.95)	1.23(1.02–1.49,p = 0.035)	0.25
Epigastralgia am	0.93(0.76–1.14,p = 0.48)	0.74(0.6–0.91,p = 0.0048)	0.13
Epigastralgia pm	0.99(0.82–1.19,p = 0.89)	0.74(0.57–0.95,p = 0.018)	0.069
Epigastralgia evening	0.78(0.58–1.06,p = 0.11)	0.78(0.63–0.97,p = 0.024)	0.99
Arthralgia am	1.03(0.84–1.27,p = 0.76)	0.94(0.81–1.1,p = 0.46)	0.49
Arthralgia pm	0.89(0.73–1.09,p = 0.26)	1(0.86–1.17,p = 0.99)	0.36
Arthralgia evening	0.98(0.82–1.19,p = 0.87)	0.97(0.71–1.34,p = 0.86)	0.94

Data were analyzed utilizing the gee model with logit link. i.e. a logistic regression was applied with the symptom (as binary) as the dependent variable and the treatment and time as covariates. Since there is more than one observation for each patient, the model also considered the possible correlations among the specified observation for the same patient (hence gee rather than a simple logistic model). The columns contain: the effect in the control arm, the effect in the IFN β-1a treated arm, and represent how the symptoms changed over time. If the OR was >1, then the frequency of the symptom increased over time, while if the OR was <1, the frequency of the symptom decreased over time. The last column contains the p-values when the OR for the historic controls was compared to the OR for the IFN-treated patients. These p-values indicate whether the changes in the symptom frequency over time differ between the two arms.

The highlighted values are significant after adjusting for multiplicity with the Hochberg’s approach. Breakdown of symptoms by time and treatment are provided in S4 Table.

## Discussion

These preliminary findings, cautiously interpreted, suggest that treatment with IFN β-1a may be associated with clearance of virus from blood, better clinical features and potentially, improved survival (summarized in S1 CONSORT Checklist). As the case fatality ratio for EVD is associated with level of blood viremia, the findings reported herein do need to be interpreted cautiously, given the difference in CT values between the controls and IFN β-1a treated patients and the limited sample size. Despite the limitations of a single arm, non-randomized study, we infer from these data that IFN β-1a treatment is worthy of further consideration for the treatment of EVD.

At the start of the outbreak, and over the ensuing months, there was considerable skepticism about the potential therapeutic effectiveness of type I IFNs. Our strategy at the outset was to evaluate the safety and efficacy of IFN treatment, hence the decision to use a non-pegylated version of IFN with a short half-life, that could be discontinued or dose-reduced, with rapid clearance, to address potential concerns of adverse events. Moreover, extensive clinical experience with IFN-α and IFN-β prepared us for potential adverse outcomes and solutions to these. The decision to undertake this trial was based on preliminary scientific pre-clinical data and, perhaps most pertinently, the absence of any approved antivirals to treat EVD. Availability and cold storage were not issues, unlike other experimental antivirals that were considered during the recent outbreak.

A unique aspect of this trial was that the onsite team was comprised exclusively of 11 Guinean nationals. Healthcare workers received, for their first time, relevant training in all operational and administrative aspects of conducting a trial. There is now in place in Guinea a team of 11 individuals, including MDs and administrative staff, who have the ability and competencies to conduct a clinical trial according to international standards.

Although the current outbreak of EVD in West Africa was declared over on January 14, 2016 by the WHO, a new case was reported in Sierra Leone in the ensuing days with identified contacts at risk of developing EVD. Therefore, there continues to exist the possibility of resurgence of EVD or emergence of a divergent strain [19]. Certainly, vaccines offer the potential to protect populations from a resurgent EBOV strain identical to that reflected in the current vaccines under evaluation, yet it remains unclear whether these vaccines offer post-exposure protection or what the duration of protection is prior to exposure. Accordingly, the need exists for antivirals that provide a broader spectrum of activity against different EBOV strains. Viewed altogether, the data support further evaluation of IFN β-1a for the treatment of EVD.

## Supporting information

S1 Protocol Interferon beta-1a protocol 01_12_15. (PDF)

S1 Table All patient analysis: Baseline characteristics and regression analysis for treatment effects on survival. (DOCX)

S2 Table Subset analysis: Baseline characteristics and regression analysis for treatment effects on survival. (DOCX)

S3 Table Matched analysis: Baseline characteristics and regression analysis for treatment effects on survival. For each patient in the IFN group an attempt was made to match with untreated patients who had a less than 5 years difference in age and less than 2 units difference in their baseline CT value. For 5 patients suitable matches were found: For IFN 02 one match, for IFN 03 one match, for IFN 04 three matches, for IFN 08 two matches and for IFN 10 three matches. For IFN 05, IFN 06, IFN 07 and IFN 09 no matches were found and they did not enter this analysis.
(DOCX)

S4 Table Effects of IFN β-1a on frequency of clinical symptoms associated with EVD. Symptoms recorded am, pm and e (evening), as indicated. With reference to asthenia pm (highlighted in grey), this symptom remained relatively unchanged over the 3 time periods examined among the historic controls receiving supporting care only (54%, 74%, 56%), and the p-value is not significant (p = 0.47). For the IFN ß-1a treated patients, the incidence of asthenia (pm) decreases over the 3 time periods (69%, 43%, 8%) with the OR in this arm of 0.65 and p<0.0001. Comparing the OR between historic controls and IFN-treated, the OR in the control arm is 1.65 fold larger than the OR in the IFN-treated arm, hence the significance recorded in the last column in Table 3.
(DOCX)

S1 CONSORT Checklist Information pertaining to historically controlled, single arm proof-of-concept trial: Interferon β-1a for the treatment of Ebola virus disease. (DOC)

S1 Excel File Details of supportive care provided to IFN-β treated patients 001–009 M~ morning. AFT~ afternoon, E~ evening.
(XLSX)
