# Correction: Interferon β-1a for the treatment of Ebola virus disease: A historically controlled, single-arm proof-of-concept trial

**DOI:** 10.1371/journal.pone.0175930

**Published:** 2017-04-12

**Authors:** The PLOS ONE Staff

There is an error in affiliation 3 for author Darren P. Baker. Affiliation 3 should be: Sanofi Genzyme, Cambridge, Massachusetts, United States of America.

There is an error in the caption for Fig 2. Please see the complete, correct Fig 2 caption here. The publisher apologizes for these errors.

**Fig 2:**
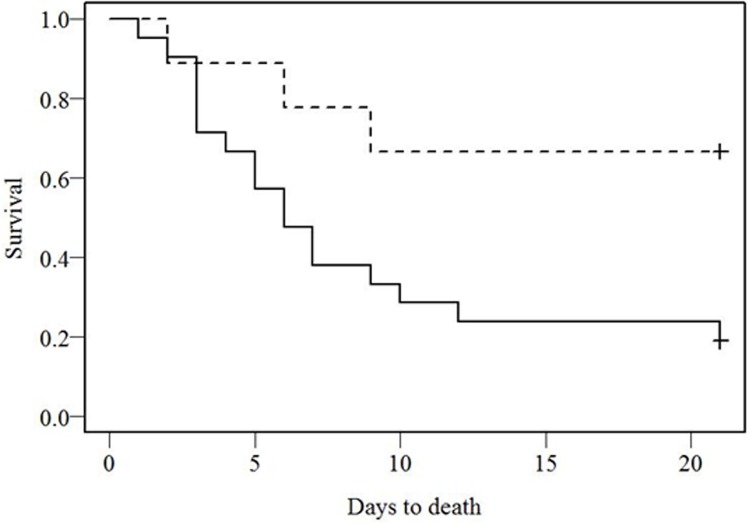
IFN β-1a treatment effect on survival of patients with EVD. Survival curves for patients with EVD receiving supportive care only (n = 21) (solid line) or supportive care plus IFN β-1a treatment (n = 9) (dashed line). Survival was calculated from the date of consent for those receiving IFN β-1a treatment and date of admission for those in the control cohort, to the date of death. Survival plots were based on Kaplan-Meier estimates and the plots were compared using the log rank test (p = 0.026).

## References

[1] Konde MK, Baker DP, Traore FA, Sow MS, Camara A, Barry AA, et al (2017) Interferon β-1a for the treatment of Ebola virus disease: A historically controlled, single-arm proof-of-concept trial. PLoS ONE 12(2): e0169255 doi:10.1371/journal.pone.0169255 28225767 10.1371/journal.pone.0169255 PMC5321269

