# Supplementary material for: Interferon β-1a for the treatment of Ebola virus disease: A historically controlled, single-arm proof-of-concept trial
Source: PLoS One. 2017 Feb 22;12(2):e0169255. doi: 10.1371/journal.pone.0169255 (PMC5321269; doi:10.1371/journal.pone.0169255)
Supplement: S1 Protocol — (PDF) [file pone.0169255.s001.pdf]

A Pilot Study to Evaluate the Safety and Efficacy of Interferon Beta-1a (IFN β-1a) in the Treatment of Patients Presenting with Ebola Virus Illness

Clinical Study Protocol
Protocol # 2014-EBOV

Sponsor-Investigator: Dr. Mandy Kader Konde, Conakry, Guinea

Collaborator: Dr. Eleanor N. Fish, University Health Network, Toronto, Ontario, Canada

Investigational product: Recombinant IFN-β-1a

Manufacturer: BiogenIdec Inc, Cambridge, MA 02142, USA

Table of Contents

Synopsis 3

1. Introduction 7
2. Background on Investigational Product 13
3. Investigators and Trial Organization 17
4. Hypothesis and Trial Objectives 17
5. Study Design 17
6. Conduct of the Study 20
7. Clinical Pharmacology of IFN β-1a 22
8. Management of Disease Progression, Adverse Events and Serious Adverse Events 24
9. Study Endpoints 26
10. Biostatistical Considerations 27
11. Data Management 28
12. Premature Discontinuation of Treatment 28
13. Conduct of the Investigation 30
14. Ethics Review and Consent 30
15. Time Frame for the Study 30
16. Analysis of Final Data 31
REFERENCES 32

Synopsis

**Product provided by:**	BiogenIdec Inc.
**Investigational Product/ Drug Product:**	Interferon beta-1a (IFN β-1a)
**Title of the Study:**	A Pilot Study to Evaluate the Safety and Efficacy of IFN β-1a in the Treatment of Patients Presenting with Ebola Virus Illness.
**Development phase:**	I/II
**Sponsor-Investigator:**	Dr. Mandy Kader Konde
**Collaborator:**	Dr. Eleanor N. Fish
**Planned Study Period:**	January/February 2015 to April/May 2015
**Primary Objective:**	To evaluate the effectiveness of IFN β-1a on viremia in peripheral blood
**Primary Endpoint:**	Viral load reduction/clearance: Reduction and/or clearance in copies/mL of viral RNA from day 1 to day 14 and, if applicable, 30 days after the last dose
**Secondary Objectives:**	To evaluate the safety of IFN β-1a in the treatment of Ebola virus disease
**Secondary Endpoints**	Occurrence and severity of adverse event; time to onset from the initial dose, duration (number of days), severity, and seriousness of solicited and non-solicited injection site reactions and systemic adverse events occurring up to 7 days after the last injection. Occurrence, nature, time to onset from the initial dose, duration, severity, relationship to injection of SAEs throughout the trial, and up to 30 days post-treatment.<br><br>The frequency of clinically important (moderate or severe) adverse events will be compared between the IFN β-1a treatment group and the group receiving usual standard of care.<br><br>The frequency of serious adverse events will be compared between the groups.
**Tertiary Objectives**	To evaluate the effect of IFN β-1a therapy on preventing Ebola virus disease progression and/or on resolution of disease.<br><br>To assess effects on disease symptoms and survival.
**Tertiary Endpoints**	Clinical improvement will be evaluated by comparing the following parameters between the groups:<br>Days in treatment centre<br>Days of symptom manifestation: hemorrhage (as IFN β-1a may cause fever, myalgia, sore throat, headache, nausea, vomiting, and diarrhea similar to that associated with ebola virus infection, these adverse events will be recorded but not used in the analysis of efficacy).

	Death – all causes for study duration
**Methodology/Trial Design:**	This is a Phase I/II, interventional trial designed to assess the safety and trends in clinical course of Ebola virus disease in patients ≥18 and <70 years of age treated with either IFN β-1a and usual supportive care, or usual supportive care only. It is associated with appropriate monitoring of therapy.
**Planned Sample Size:**	Pilot: 30-50
**Treatment and Specimen Collection Schedules and Duration of Follow-up:**	Subcutaneous injection of IFN β-1a daily for up to 10 days.<br>Up to 11 blood samples will be drawn on days 1-10 and one month after discharge from the treatment centre.<br>Subjects will be followed for the duration of their stay in the treatment centre.<br>From first patient enrolment to last patient last visit, it is expected that the study will last 4 months.
**Investigational Product:**	Recombinant IFN β-1a; an injectable sterile suspension with licensed IFN-β provided in single use syringes
Form:	Sterile, clear, colorless preservative-free liquid, single-use syringe
Composition:	Each 0.5 mL of 60 microgram (mcg)/mL of IFN β-1a in a pref-filled glass syringe contains 30 mcg of IFN β-1a, 0.79 mg of sodium acetate trihydrate USP; 0.25 mg of glacial acetic acid USP; 15.8 mg of arginine hydrochloride USP; and 0.025 mg Polysorbate 20 in water for injection, USP to a pH of about 4.8.
Route:	Subcutaneous (sc) injection - undiluted
Lot Number:	V32482
**Inclusion Criteria:**	Only potential patients fulfilling all of the following criteria are eligible for trial enrolment:<br>1. Able to provide informed consent. Substitute decision maker may provide informed consent in cases where the patient is ill and unable to provide informed consent.<br>2. Aged ≥18 years and <70 years of age on the day of inclusion.<br>3. In the treatment centre.<br>4. Suspect, probable or confirmed Ebola virus infection as per case definitions.<br>5. Symptom onset <4 days.<br>6. Able to comply with trial procedures.<br>The treating physician is responsible for recommending and for initiating therapy with IFN β-1a through the Special Access/Compassionate Release Program.
**Exclusion Criteria:**	A potential subject fulfilling any of the following criteria is to be

	excluded from the randomized controlled trial enrolment:<br>1. Known hypersensitivity to interferon beta preparations<br>2. Pregnancy (the use of IFN β-1a is associated with arbortifacient effects in rhesus monkeys and may cause miscarriage in humans)<br>3, Chronic liver disease with synthetic dysfunction and/or decompensation (ascites, hepatic encephalopathy, history of bleeding oesophageal or gastric varices)<br>4. Moderate to severe congestive heart failure (CHF) - grade III or IV left ventricular function<br>5. Previous history of serious psychiatric illness (e.g. psychosis, suicide ideation and/or attempt, depression requiring hospitalization)*<br>6. History of severe or active autoimmune disease (systemic lupus erythematosus, thyroiditis, vasculitis).<br><br>* These precautions will be reconsidered on a case-by-case basis for patients who are ill to weigh the risks and benefits of IFN β-1a therapy in the presence of these underlying conditions.<br><br>The exclusion criteria can be reviewed by the treating physician to assess the potential risks of therapy through the Special Access/Compassionate Release Program.
**Precautions and temporary contraindications**	The following should be considered precautions or temporary contraindications since these conditions can be exacerbated by administration of IFN β-1a. However, if they can be corrected and/or controlled prior to and during administration of IFN β-1a, they will not result in exclusion of the potential participant:<br>1. Thrombocytopenia (platelets <25,000/μL or bleeding disorder contraindicating SC injection<br>2. Neutropenia (Absolute neutrophil count <1000/μL)<br>3. Anemia (hemoglobin <70g/L)<br>4. Uncontrolled seizures<br>5. Moderate to severe uncontrolled hypertension<br>6. Clinical gout<br><br>These precautions should be reviewed by the treating physician to assess the potential risks of therapy through the Special Access/Compassionate Release Program.
**Statistical Methods and Analyses**	Endpoints will be described and compared between baseline and day 10, for the treatment group using cross-tabulation tables and Fisher's Exact test for categorical variables, t-test for normally distributed continuous variables, and Wilcoxon non-parametric test for non-normally distributed continuous or ordinal variables. Data collected will be compared with a retrospective analysis of age-matched Ebola-infected individuals who presented with similar symptoms (and viral loads) and who did not receive IFN β-1a.

	Safety variables such as fever will be considered in three different formats:<br>1. As ordinal, representing various levels of severity of the AE. For instance, fever will be described as “none”, “mild”, “moderate” or “severe”.<br>2. As binary, considering only the presence vs. the absence of the AE.<br>3. As binary, considering the most serious expression of the AE. For instance by grouping “moderate” and “severe” together.<br>Separate analyses for time periods of 0-3, 4-7 and 8-10 days will be conducted. For each of these time periods the most severe report of the AE for each participant will be counted.<br><br>Adverse Events and Serious Adverse Events will be considered in two ways: presence of any event and presence of any event related to the treatment.<br><br>Viral clearance and symptom endpoints will be analyzed as binary variables for detection (presence) or non-detection (absence) as well as continuous variables where applicable (e.g.; quantitative RT-PCR measurements.

1. Introduction

1.1 Background on Ebola Virus Infection

Clinical Ebola hemorrhagic fever is characterized by onset of fever, headache, myalgia, fatigue and gastrointestinal distress, 2-21 days post exposure to virus. Immune responses play a critical role in Ebola virus (EBOV) pathogenesis: infection is associated with an early loss of lymphocytes and down-regulation of interferon production and actions. There is subsequent dysregulation of coagulation, leading to clinically important hemorrhage in a minority of patients. The genus *ebolavirus* includes 5 different viruses: Sudan virus, Tai Forest virus, EBOV, Reston virus and Bundibugyo virus. EBOV has the highest fatality rate – 57-90%. Data from human infections with EBOV are limited, but the evidence from previous outbreaks suggests that the innate immune response is predictive of outcome. Specifically, an early and transient pro-inflammatory cytokine response, including IFN-α, is associated with survival, yet a sustained and increasing pro-inflammmtory cytokine response, with dendritic cell infection that abrogates the IFN response, is associated with death (1, 2). Notably, in the absence of a robust innate immune response, including IFN- α activation of dendritic cells, the adaptive immune response (B and T cell activation) is severely impaired, leading to failure of control of viral replication and poor outcome.

1.2 Epidemiologic situation as of November 19, 2014 (WHO data)

In the context of this outbreak, this is the first outbreak in West Africa, therefore there is a lack of understanding within local communities, lack of experience among health care workers and limited capacity for rapid response. There is a high level of community exposure, a consequence of household care and customary burial procedures, leading to fear, panic and some resistance to proposed response measures. Close community ties across border areas are having an impact on care-seeking behaviors and contact tracing. The magnitude of the spread of this outbreak in the 3 most affected countries, as of November 19, 2014, is in rural areas and in large cities, (see Table 1), and requires an enormous commitment of resources and robust sustained response capacity.

There is no vaccine or specific approved treatment for EBOV disease currently available. At the present time, patients are only provided with usual supportive care on arrival at treatment centres . There are promising data related to novel vaccines and potential antiviral drugs, derived from *in vitro* and pre-clinical animal models of EBOV infection, yet these have not been evaluated for safety and efficacy in humans.

In the current context, the WHO has recommended that it is ethical to offer unproven interventions (with unknown efficacy and unknown adverse events) as potential treatments or prevention strategies. Any therapeutic intervention must be administered with informed consent and freedom of choice, the decision having been made following a thorough risk-benefit assessment, and respecting the confidentiality and dignity of the patient. In addition, there is a moral obligation to collect and share all data generated, to understand the safety and efficacy of any intervention, including a moral duty to evaluate any intervention in the best possible clinical trial feasible, and to inform future interventions.

Country	Cumulative cases (Conf-Prob)	Cumulative deaths
Guinea	2047	1214
Liberia	7082	2963
Sierra Leone	6190	1267

Table 1

1.3 WHO Working Group to Evaluate Lead Experimental Therapeutic Intervention Candidates: outcomes

In late August 2014, a WHO-initiated Working Group was assembled to consider the various experimental therapeutic intervention candidates for EBOV disease. Early during the deliberations the decision was made to evaluate only those candidates that have been evaluated in pre-clinical rodent and nonhuman primate models of EBOV disease.

Vaccine candidates for general use pre-exposure and post-exposure prophylaxis included the vesicular stomatais virus/Ebola Zaire glycoprotein vaccine (NewLink/Public Health Agency of Canada), requiring -80˚ to -20˚C storage, for which there are currently 800 doses available and has been evaluated in nonhuman primates. A number of other candidate vaccines were considered, lacking nonhuman primate efficacy data, GMP manufactured product and or other nonclinical studies. Notably, the Crucell ChAd3 adenovirus vaccine is poised to be evaluated in Phase 1 studies in the Gambia and Mali in late September 2014, with projected availability of 15,000 doses of vaccine for Phase 2 studies in December, 2014.

Candidate antiviral therapeutic interventions were also evaluated. These included antiviral agents that may target the virus directly, may target a host function required for the viral life cycle and/or may augment host defenses. With the exception of USFDA-approved drugs that are being considered for repurposing as antivirals against EBOV disease, all of the other antiviral agents considered are under development. Actual treatment doses and regimens are uncertain at the current time and the mechanism of action of individual agents will dictate their temporal usage (early vs. late stage disease).

1.4 Candidate Therapeutic Interventions

ZMapp (LeafBio) is produced in tobacco plants, is a triple monoclonal antibody cocktail, with a mechanism of action that neutralizes the virus and kills infected cells. Nonhuman primate studies revealed 100% treatment efficacy when treatment was initiated 5 days after virus infection. There are no controlled human safety data and no product currently available.

Human convalescent serum There is limited nonhuman primate efficacy study data that suggest passive transfers may provide partial protection. An ISARIC protocol for convalescent plasma collection and administration is under development. The availability and feasibility of administration of convalescent serum is location-dependent, i.e. available infrastructure to collect, process, and screen.

Antibody infusions Equine F(ab)2 (Fabentech, France) and Transchromosomic cattle human polyclonal antibody (Sanford Applied Biologics, USA) are under development, with projected availability in 6 months.

TKM-100802 (Tekmira Pharmaceuticals) is a cocktail of 2 small interfering RNAs (siRNAs) in lipid nanoparticle, targeting the viral proteins VP35 and L Polymerase, thereby silencing the viral genes and preventing viral replication. In nonhuman primate studies there was evidence of 83% treatment efficacy when initiated 48 hours after virus infection, and 67% treatment efficacy when initiated 72 hours post infection. Phase 1 single ascending dose studies have been completed. Storage of drug is at room temperature. Currently there are 30 treatment courses available with a projected 54 treatment courses to be available in 3.5 months and 490 in 6 months.

AVI-7537 (Sarepta) is a phosph morpholino oligonucleotide that blocks viral protein production. Nonhuman primate studies showed 60-80% efficacy when treatment was initiated at 1 hour post infection. Phase 1 single ascending doses studies have been completed. Treatment course is 14 days with room temperature storage of drug. Potential availability of 24 treatment courses by mid-October is projected.

Favipiravir (T-705) (Medivector/Toyma/Fuji) is a small molecule, viral polymerase inhibitor that has been conditionally approved in Japan for influenza virus infection. Published *in vitro* and mouse model studies suggest that the drug is anywhere from 10-60 fold less effective against EBOV than influenza virus. Pilot nonhuman primate studies are underway with anticipated higher dosing. Up to 15,000 treatment courses are currently available, with a projected 100,000 treatment courses to be available in 3 months. It is anticipated that 90 tablets are required over 5 days for human treatment course. Given the high incidence of nausea, vomiting and diarrhea in EBOV-infected individuals, this oral bioavailability presents a significant concern.

BCX4430 (BioCryst Pharmaceuticals, Inc.) is a small molecule, viral polymerase inhibitor. Efficacy has been demonstrated *in vitro* and in mouse models of EBOV disease. Nonhuman primate studies are anticipated to begin shortly. A preclinical IND submission is anticipated in November 2014, with potentially 2000 treatment courses projected to be available in March 2015.

Brincidofovir (CMX001) (Chimerix) is a small molecule, viral polymerase inhibitor that has demonstrated *in vitro* antiviral activity against filoviruses. Because of metabolism differences its evaluation in Macaque nonhuman primates is precluded. Phase 3 studies in >1000 trial subjects have been conducted against CMV and adenovirus infections. Potentially there are 3,500 treatment courses currently available. Oral bioavailability given nausea, vomiting, and diarrhea is a concern for the 6-10 tablet treatment course.

Coagulation related interventions Recombinant nematode anti-coagulant protein C2 that blocks Factor X cleavage has completed Phase 2 trials. The drug exhibits 33% efficacy when initiated 24 hours after infection in nonhuman primates. 100 doses are currently availability, dependent on ongoing stability testing. Drotrecogin alfa was approved for the treatment of sever sepsis, but has been withdrawn. Nonhuman primate studies showed 18% treatment efficacy when administered <6 hours after infection. Availability is unknown.

1.5 Repurposing Approved Drugs

Given the poor availability and logistics/feasibility surrounding administration of some of the candidate interventions evaluated above, the repurposing of approved drugs was evaluated. Advantages include availability and clinical experience with these drugs. Briefly, these included Clomiphene/Toremifene, selective estrogen receptor modulators that may have an effect on late viral entry and have some demonstrated *in vitro* efficacy, also affording 90% survival when administered 1 hour after infection of mice. However, nonhuman primate studies identified ocular disturbances that resulted to studies being suspended while these adverse events are investigated. *In vitro* screens of libraries of drugs have identified a number of candidates, including ion channel inhibitors, kinase inhibitors, anti-parasitic drugs and anti-inflammatory drugs, however additional studies are needed to further evaluate the potential of these interventions in EBOV disease.

1.5.1 Interferon

Interferons (IFNs) are classified as type I (IFNs-α, -β, -ω, -τ -κ, -ε, ξ, type II (IFN-γ) or type III (IFNs-λ: IL-28A, IL-28B, IL-29). Type I IFNs mediate diverse biological effects, including the largely species-specific but cell type-independent antiviral responses, and several cell type-restricted responses of immunological relevance. IFNs modulate both T and B lymphocyte responses by promoting the proliferation of memory T cells, promoting the differentiation of Th1 cells, inducing IFN-γ secretion from T cells, and promoting isotype switching in B cells and differentiation into plasma cells. IFNs-α/β upregulate MHC class I. Moreover, IFNs activate macrophages, activate and enhance the cytotoxicity of NK cells, and regulate the maturation and terminal differentiation of dendritic cells. IFNs have widespread potential as therapeutic agents for the treatment of viral infections, and are currently administered for chronic hepatitis B and C (HCV) infections. IFNs inhibit viral infection by preventing viral entry into target cells and by blocking different stages of the viral replicative cycle for different viruses. Type I IFNs have a critical role in linking the innate and adaptive immune responses to viral challenge. IFNs-α/β regulate the activities of other cytokines and their receptors, including IFN-γ, IL-1, IL-1 receptor, IL-2, IL-3, IL-8, TNF-α, IL-18 etc, and chemokines such as CCL3, CCL4, CCL5, and the chemokine receptor, CCR5.

IFN-α/β expression occurs as the earliest non-specific response to viral infection, mediated by activation of microbial pattern recognition receptors including TLRs and cytoplasmic receptors RIG-I/MDA5, preceding other cytokines. Indeed, viruses have evolved immune evasion strategies specifically targeted against an IFN response, confirming the importance of IFNs as antivirals.

This immune evasion strategy is relevant when one considers IFN treatment for EBOV infection (3, 4). Experimental data indicate that EBOV proteins VP24 and VP35 inhibit host cell systems that lead to IFN production and also inhibit events associated with an IFN response. EBOV VP35 effectively blocks IFN-α/β production by either RIG-I or MDA5 (5). Indeed, specific effector molecules of an IFN response have been shown to directly inhibit EBOV entry into cells and release of viral progeny from cells, effectively preventing viral spread. Treating EBOV infected individuals with IFN may therefore override the inhibitory effects of EBOV infection on IFN *production*, but the challenge remains to administer treatment early post-exposure, before the viral burden is such that IFN responsiveness would be disabled.

IFN-α2 has has been approved by the USFDA for the treatment of AIDS-related Kaposi's Sarcoma, and hepatitis B. In combination with ribavirin, different IFN-α2 preparations and Infergen (IFN-alfacon-1) have been approved by the USFDA for hepatitis C infection (and are currently being evaluated in HIV infection). Estimates of 40% sustained virologic response against HCV genotype 1 and 70% against genotypes 2 and 3, using the pegylated IFN-αs in combination with ribavirin, provide evidence for the therapeutic benefits of IFNs-α as antivirals. IFN-α has also been shown to have anti-inflammatory effects by decreasing cell proliferation and increasing circulating levels of TNF-α-receptor. Thus IFN-αs have been demonstrated clinically to have a role in treating viral diseases.

Evidence for the utility of IFNs in Ebola virus therapy

1. *In vitro* inhibition of EBOV replication. See Figure 1.
2. Inhibition of type 1 IFN signaling increases filovirus severity (3,4,6). Notably, when rhesus macaques were administered an antibody against one of the IFN receptor subunits, IFNAR1, on days 2 and 5 after virus inoculation, there was increased viremia and earlier progression to death (6).
3. IFN β-1a therapy in rhesus macaques: at 10.5 μg/kg starting at 18 hours after virus inoculation and continuing to 9 days, prolonged time to death from EBOV. 35 μg/kg starting 1 hour post virus inoculation and daily for 14 days, led to survival of 1/3 from MARV infection (6).
4. IFN-α2b therapy in cynomologous macaques: at 2 x 10^7 IU/kg im starting at 18 hours after virus inoculation and daily, reduced viremia (7).
5. Adenovirus expressing IFN-α enhances the protective efficacy of mAbs in NHP when administered after onset of symptoms (8).
6. Unpublished *in vitro* evidence for potent antiviral activity against EBOV (Bavari, S. (USAMARIID); Hensley S. (NIAID), Geisbert, T. (UTMB, Galveston), Rubins, K., (Whitehead Institute).

7. Guinea pig studies providing evidence that adenovirus expressing IFN-α2 days post-infection reduces viremia (9).
8. Examination of serum samples from infected individuals during the 2000 outbreak of Sudan EBOV in Uganda revealed that surviving patients had significantly higher levels of IFN-α within the first few days of the onset of critical illness (1).
9. Evidence that circulating levels of IFNs-α/β are only detectable 4 days after virus inoculation of cynomologous macaques, likely delayed because of virus infecting dendritic cells (10). This delay in the IFN-inducible immune response likely contributes to the virulence of infection.
10. Availability of stocks of FDA-approved IFNs.

Figure 1 Dose response of IFN-α in EBOV (HeLa) assay

AC_{50}=119 pg/mL

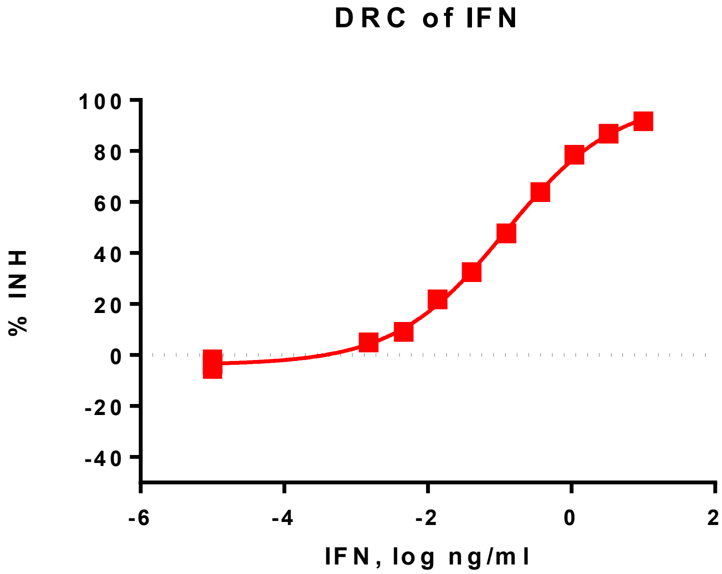

Notably, in all the nonhuman primate studies where either an IFN-β or an IFN-α or the adenoviral vectored-Infergen gene was administered, there are 3 considerations that may account for the limited therapeutic benefit that was observed: 1. The challenge dose of EBOV was 1000 PFU by intramuscular route of inoculation. Both the route of inoculation and the significantly high lethal challenge dose may distinguish course of infection in the nonhuman primates compared with the current human EBOV disease. Of note, the illness in nonhuman primates is more rapid in progression and more lethal than that seen in humans. 2. IFN dosing. In contrast to current dosing for therapeutic efficacy against HCV (and for multiple sclerosis), doing regimens employed for these published nonhuman primate studies were of the order of 10-100 fold greater (mg/kg). This raises the issue of invoking adverse events that may undermine the potential therapeutic benefit of dosing that might reflect what will be used in humans i.e. survival may have been compromised in these studies. 3. Differences in the innate and adaptive immune responses between nonhuman primates and humans. While some of the rhesus IFN-α subtypes are homologous to the corresponding human sequences (IFN-α1/13, α2, α6, α8, α14, and α16), 7 others have a partial homology to human IFN-α4, α16, or α21, and have been termed IFN-α23 to IFN-α29 (11). TLR7 and TLR9 agonists mediate distinct type I IFN responses in humans and nonhuman primates *in vitro* and *in vivo* (12). Differences in Th1 and Th2 cytokine profiles between humans and nonhuman primates also exist (13). Nonhuman primates are more sensitive to vaccines than humans (14).

2. Background on Investigational Product

IFN β-1a is a 166 amino acid glycoprotein with a molecular weight of approximately 22,500 Daltons. It is produced by recombinant DNA technology using genetically engineered Chinese Hamster Ovary cells into which the human interferon beta gene has been introduced.

The amino acid sequence of IFN β-1a is identical to that of natural human IFN β.

Using the World Health Organization (WHO) International Standard for Interferon, the study drug has a specific activity of approximately 200 million international units of antiviral activity per mg of IFN β-1a determined using an *in vitro* cytopathic effect bioassay using lung carcinoma cells (A549) and Encephalomyocarditis virus (EMCV).

30 mcg contains approximately 6 million international units of antiviral activity using this method. The activity against other standards is not known. Comparison of the activity of BiogenIdec's IFN β-1a with other IFNs β is not appropriate because of differences in the reference standards and assays used to measure activity.

2.1 Rationale for Choice of IFN β Preparation

1. Data from published and unpublished nonhuman primate studies suggest that IFN β-1a treatment may be more effective in reducing viremia and prolonging survival than IFN-α.
2. Availability of pre-filled syringes of IFN β-1a.

In addition, the following supports the choice of IFN β-1a:

Like other members of the Type I IFN family, IFN β-1a has been shown to protect cells from virus infection, as well as showing efficacy in HCV-infected individuals (15). Moreover, while IFN-α has been used to treat HCV in Europe and the US, natural, non-recombinant IFN-β has been used extensively in Japan for this indication, and there is data to suggest that in HCV, where both IFN-α and IFN-β have been used to treat the disease, that the safety and tolerability of IFN-β is more favorable than for IFN-α (16, 17). In addition to the known antiviral activity of IFN β-1a in HCV and its antiviral activity in Ebola-infected rhesus macaques (6), AVONEX has a favorable safety and tolerability profile in MS patients with approximately 1.9 million person years of exposure worldwide (BiogenIdec Inc., data on file).

2.2 Clinical Experience with AVONEX in multiple sclerosis

The clinical effects of AVONEX in patients with relapsing forms of multiple sclerosis (MS) were studied in two randomized, multicenter, double-blind, placebo-controlled studies in patients with MS (Studies 1 and 2). Safety and efficacy of treatment with AVONEX for over 10 years has been studied.

In Study 1, 301 patients received either 30 mcg of AVONEX (n=158) or placebo (n=143) by intramuscular injection once weekly. Patients received injections for up to 2 years, and continued to be followed until study completion. Two hundred eighty-two patients completed 1 year on study, and 172 patients completed 2 years on study. There were 144 patients treated with AVONEX for more than 1 year, 115 patients for more than 18 months and 82 patients for 2 years.

All patients had a definite diagnosis of multiple sclerosis of at least 1 year duration and had at least 2 exacerbations in the 3 years prior to study entry (or 1 per year if the duration of disease was less than 3 years). At entry, study participants were without exacerbation during the prior 2

months and had Kurtzke Expanded Disability Status Scale (EDSS) scores ranging from 1.0 to 3.5. The EDSS is a scale that quantifies disability in patients with MS and ranges from 0 (normal neurologic exam) to 10 (death due to MS). Patients with chronic progressive multiple sclerosis were excluded from this study.

The primary outcome assessment was time to progression in disability, measured as an increase in the EDSS score of at least 1 point that was sustained for at least 6 months. An increase in EDSS score reflects accumulation of disability. This endpoint was used to help distinguish permanent increase in disability from a transient increase due to an exacerbation.

The time to onset of sustained progression in disability was significantly longer in AVONEX-treated patients than in placebo-treated patients in Study 1 ($p = 0.02$). The percentage of patients progressing by the end of 2 years was 35% for placebo-treated patients and 22% for AVONEX-treated patients. This represents a 37% relative reduction in the risk of accumulating disability in the AVONEX-treated group compared to the placebo-treated group.

The distribution of confirmed EDSS change from study entry (baseline) to the end of the study was examined. There was a statistically significant difference between the AVONEX and placebo groups in confirmed change for patients with at least 2 scheduled visits ($p = 0.006$).
The rate and frequency of MS exacerbations were secondary outcomes. For all patients included in the study, irrespective of time on study, the annual exacerbation rate was 0.67 per year in the AVONEX-treated group and 0.82 per year in the placebo-treated group ($p = 0.04$).

AVONEX treatment significantly decreased the frequency of exacerbations in the subset of patients who were enrolled in the study for at least 2 years (87 placebo-treated patients and 85 AVONEX-treated patients; $p = 0.03$).

Gadolinium (Gd)-enhanced and T2-weighted magnetic resonance imaging (MRI) scans of the brain were obtained in most patients at baseline and at the end of 1 and 2 years of treatment. Secondary outcomes included Gd-enhanced lesion number and volume, and T2-weighted lesion volume. Gd-enhancing lesions seen on brain MRI scans represent areas of breakdown of the blood brain barrier thought to be secondary to inflammation. AVONEX-treated patients demonstrated significantly lower Gd-enhanced lesion number after 1 and 2 years of treatment than placebo-treated patients ($p < 0.05$). The volume of Gd-enhanced lesions showed similar treatment effects in the AVONEX and placebo groups ($p < 0.03$). Percentage change in T2-weighted lesion volume from study entry to Year 1 was significantly lower in AVONEX-treated than placebo-treated patients ($p = 0.02$). A significant difference in T2-weighted lesion volume change was not seen between study entry and Year 2 in the AVONEX and placebo groups.

The exact relationship between MRI findings and the clinical status of MS patients is unknown. The prognostic significance of MRI findings in these studies has not been evaluated.

In Study 2, 383 patients who had recently experienced an isolated demyelinating event involving the optic nerve, spinal cord, or brainstem/cerebellum, and who had lesions typical of multiple sclerosis on brain MRI, received either 30 mcg of AVONEX ($n = 193$) or placebo ($n = 190$) by intramuscular injection once weekly. Patients were enrolled into the study over a two- year period and followed for up to three years or until they developed a second clinical exacerbation

in an anatomically distinct region of the central nervous system.
In Study 2, the primary outcome measure was time to development of a second exacerbation in an anatomically distinct region of the central nervous system. Time to development of a second exacerbation was significantly delayed in AVONEX-treated compared to placebo-treated patients ($p = 0.002$). The Kaplan-Meier estimates of the percentage of patients developing an exacerbation within 24 months were 39% in the placebo group and 21% in the AVONEX group. The relative rate of developing a second exacerbation in the AVONEX group was 0.56 of the rate in the placebo group (95% confidence interval 0.38 to 0.81).

2.3 Safety Considerations of IFN β-1a

2.3.1 Potential Risks

Depression, Suicide, and Psychotic Disorders.

Hepatic Injury: monitor liver function tests; monitor patients for signs and symptoms of hepatic injury; consider discontinuation of IFN β-1a if hepatic injury occurs.

Anaphylaxis and Other Allergic-Reactions: Discontinue if occurs.

Congestive Heart Failure: monitor patients with pre-existing significant cardiac disease for worsening of cardiac symptoms.

Decreased Peripheral Blood Counts: monitor complete blood count.

Autoimmune Disorders: consider discontinuation of IFN β-1a if new autoimmune disorder occurs.

The most common adverse reactions (at least 5% more frequent on AVONEX than on placebo) were flu-like symptoms including chills, fever, myalgia, and asthenia.

2.3.2 Procedures to Minimize Risks

Alcohol swabbing of the skin prior to venipuncture and IFN β-1a injection, and use of sterile disposable needles and syringes will minimize the risk of infection. Applying pressure over needle puncture sites to minimize bruising should also be carried out. Exclusion of patients who have previously experienced a severe allergic reaction to IFN β preparations, and since participants will probably remain in the treatment centres for the duration of treatment, trained personnel will be available in case for allergic reactions post-injection.

For the first ten (10) ebola-infected subjects to receive IFN β-1a, a staggered administration schedule will be used to minimize the effect of any unforeseen serious adverse events in this patient population. The second patient will not be administered IFN β-1a until the first subject has received five (5) doses. Likewise, the third subject will not be administered IFN β-1a until the second subject has received 5 doses. If, in the opinion of the treating physician and the Special Access Program in Conakry, Guinea, no unforeseen serious adverse events are observed five (5) days after the tenth subject has received IFN β-1a, more than one of the remaining

subjects enrolled/to be enrolled in the study may receive their first dose of IFN β-1a on the same day.

2.3.3 Potential Benefits

This study offers a potential for antiviral therapy for patients with EBOV disease. Although IFN β-1a has not been administered to patients with EBOV infection, the clinical experience of IFN β-1a with another virus (HCV) demonstrates a favorable safety profile. Thus the major benefit of this study may be in helping patients with more severe EBOV disease to recover more rapidly, to prevent complications, and to prevent death.

The risks of this study are acceptable considering the known safety profile of the investigational product and the potential benefit to the patients is high, yielding a favorable risk-benefit ratio.

3. Investigators and Trial Organization

The trial will be conducted by investigators in Conakry, Guinea. Centres will be selected based on interest to participate and ability to conduct this trial.

4. Hypothesis and Trial Objectives

The study hypothesis is that IFN β-1a therapy will reduce viral load in Ebola-infected subjects, that IFN β-1a is safe to use in these individuals, and that the complications associated with EBOV infection can be prevented with IFN β-1a as a consequence of inhibiting viral replication.

The primary objective of this pilot study is to evaluate the effectiveness of IFN β-1a on viremia in peripheral blood of Ebola-infected subjects

The secondary objective is to evaluate the safety of IFN β-1a in Ebola-infected subjects

The tertiary objective is to determine whether IFN β-1a can prevent Ebola viris disease progression and/or on resolution of disease

5. Study Design

This study is single-arm clinical trial with a pre- and post- study design. The study will include patients who are admitted with EBOV infection to a treatment centre. If a patient meets the inclusion criteria and gives informed consent (or informed consent is given by a substitute decision maker who is responsible for the patient due to the patient's illness), the patient will be enrolled into the study i.e. the first 30-50 eligible patients will be enrolled. Patients who receive IFN β-1a will be treated through the Special Access/Compassionate Release Program (consent to treatment through their treating physician) administered in Conakry, Guinea. This part of the study will collect data retrospectively, including results from research testing of (residual/to be discarded) blood samples. The study team can make available the systematic data collection forms to the treating physician to help in monitoring of adverse events and effect of therapy, enabling more specific reporting to the Special Access Program administered in Conakry, Guinea by the treating physician for patients receiving IFN β-1a.

As noted above, retrospective data collection will include patients with EBOV disease who do not participate in this clinical trial. Data from IFN β-1a-treated patients will be matched with data from patients who did not receive IFN β-1a, based on age, sex, estimated days post-onset of symptoms, and viral load (if available). This data collection is similar to that proposed in this protocol. Also, this protocol proposes to test blood collected clinically (residual or to be discarded) for comparisons between the groups in this interventional study.

Pre- and post- Clinical Trial:
The authority to approve a treating physician's access to IFN β-1a for treatment of patients under the Special Access Program, is based on a medical decision for treatment and a discussion between the treating physician and the patient (or the patient's substitute decision maker). The collection of safety and efficacy data for monitoring by the treating physician and reporting to Dr. Kader Konde can be done based on the study case report. This will facilitate reporting by the treating physician and allow more specific and consistent reporting to Dr. Kader Konde.

This component of the study is to provide the ability to have special access to IFN β-1a if indicated clinically while providing consistent monitoring of adverse events and effect of therapy. Considering that some patients may continue to deteriorate despite antivirals or that they may be infected with an antiviral resistant strain, IFN β-1a will be made available to treating physicians for patients for whom the treating physician believes that there may be a benefit to giving IFN β-1a. Consent for treatment with IFN β-1a would be obtained by the treating physician. Monitoring of treatment will be important to ensure that any safety concerns are identified. Monitoring will include collecting information from the patient's chart as well as some research testing of residual samples which would otherwise be discarded. A template of the informed consent form is attached. The comparison group will be the group of patients who have EBOV disease and do not receive IFN β-1a.

Of note, it is recognized that there will likely be missing data on study participants due to retrospective data collection. Collection of data will be based on the clinical course and interventions required in the care of these patients.

5.1 Patient Selection and Recruitment
Patients with suspected, probable or confirmed EBOV infection will be identified as they are admitted to the participating treatment centre.

The goal will be to identify potential participants as early as possible to provide time for the informed consent process and to follow the course of their disease and avoid delays in IFN β-1a therapy. It is anticipated that many of the potential participants who will receive IFN β-1a will be ill, and considering that early therapy with IFN β-1a may be more beneficial than later intervention, delays in administration could be detrimental to the participant.

5.2 Definitions

The following definitions will be used in the conduct of this study:

A. Case definitions for Suspect or Probable Ebola virus infection:

Clinical Criteria for Ebola virus infection

- One or more clinical findings of illness associated with a history of at least one of the following symptoms:
  - Temperature of >38º C (100.4º F) and/or
  - Myalgia and/or
  - Fatigue and/or
  - Headache and/or
  - Sore throat and/or
  - Nausea or vomiting
  - Diarrhea
  - Hemorrhage

Confirmed Case: Ebola virus disease which meets the clinical criteria with laboratory confirmation for the presence of EBOV by PCR (or qRT-PCR) for the presence of viral genome and/or acute/convalescent serology.

5.3 Inclusion Criteria

Only potential patients fulfilling all of the following criteria are eligible for enrolment in this interventional trial.

1. Able to provide informed consent. Substitute decision maker may provide informed consent in cases where the patient is ill and unable to provide informed consent.
2. Aged ≥18 years and <70 years of age on the day of inclusion.
3. Is admitted to the treatment centre.
4. Suspect, probable or confirmed EBOV infection as defined above.
5. Symptoms onset <4 days
6. Is able to comply with all trial procedures.

5.4 Exclusion Criteria

A potential patient fulfilling any of the following criteria is to be excluded from study enrolment:

1. Known hypersensitivity to interferon beta preparations
2. Pregnancy (the use of IFN β-1a is associated with arbortifacient effects in rhesus monkeys and may cause miscarriage in humans)
3. Chronic liver disease with synthetic dysfunction and/or decompensation (ascites, hepatic encephalopathy, history of bleeding esophageal or gastric varices)
4. Moderate to Severe congestive heart failure, grade III or grade IV LV function
5. Previous history of serious psychiatric illness (e.g., psychosis, suicide, depression requiring hospital admission)

6. History of severe autoimmune disease (systemic lupus erythematosus, thyroiditis, vasculitis)

For this interventional study, the exclusion criteria can be reviewed, by the treating physician, to assess the potential risks of therapy.

5.5. Precautions and Temporary Contraindications

The following should be considered precautions or temporary contraindications since these conditions can be exacerbated by administration of IFN β-1a. However, if they can be corrected and/or controlled prior to and during administration of IFN β-1a, they will not result in exclusion of the potential participant. If they cannot be corrected and/or controlled prior to or during administration of IFN β-1a, they will become exclusion criteria.

1. Thrombocytopenia (platelets <25,000/μL or bleeding disorder contraindicating SC injection. Patients may receive platelet transfusions
2. Neutropenia (Absolute neutrophil count <1000/μL)
3. Anemia (hemoglobin <70g/L) (patients may receive PRBC transfusions)
4. Uncontrolled seizures (expected to be controlled in hospital)
5. Moderate to severe uncontrolled hypertension (expected to be controlled in hospital)
6. Clinical gout

* These precautions will be reconsidered on a case-by-case basis for patients who are ill to weigh the risks and benefits of IFN β-1a therapy in the presence of these underlying conditions.

For this observational study, the precautions can be reviewed by the treating physician to assess the potential risks of therapy.

6. Conduct of the Study

This will be a study undertaken in Guinea.

6.1 Informed Consent Process

Clinical Trial:
All participants (or substitute decision makers) must understand and sign the informed consent document. The Guinean physicians and associated health workers will play an important role in addressing cultural and ethical sensitivities associated with the treatment protocol, and the Guinean authorities have indicated they will assume responsibility for disseminating information about the nature of clinical trials aimed at treating Ebola virus disease.

The observational part of the study will collect data retrospectively, and results from research testing of (residual/to be discarded) blood samples and respiratory secretions.

The comparison group for this part of the study will in part be the population that may be included in an existing epidemiologic study for which there is a waiver of consent. For patients

in the comparison group who were not enrolled in such a study where waiver of consent was applied, all reasonable efforts will be made to obtain informed consent where that individual's data will be used. However, it should be noted that obtaining informed consent from a non-IFN β-1a-treated patient who survived the ebola infection and left the clinic may be difficult to obtain, as it may be from the relatives of those non-IFN β-1a-treated patients who died following ebola infection. Retrospective data collection and testing will be as consistent as possible with the clinical trial data collection form, depending on data available from medical records. Research testing will be performed on the blood and secretions samples (residual or to be discarded).

6.2 Initial Evaluation and Baseline Investigations:
Once the informed consent is signed, and prior to initiation of therapy, eligibility will be confirmed. Some of the data (e.g. demographics) may be collected retrospectively from the patients' chart (source document). Demographics will be collected, the participant will undergo evaluation to review the medical history (will be recorded from the chart and/or obtained from the participant or family members) and a physical examination will be performed by a study physician or one of the treating physicians (chart). These will be recorded in the case report form. The patient's current clinical status will also be documented.

Pre-study screening and baseline investigations will occur, preferably on the same day as signature of the Informed Consent, but definitely prior to initiation of treatment with IFN β-1a, and will be stated as performed on day 1 of hospitalization and day 'X' of illness. Follow-up data will also be recorded on this basis for comparison between relevant time points amongst participants (treated or not with IFN β-1a). It is expected that most eligible participants will be treated within 24 hours of being admitted to the treatment centre. Earlier therapy with IFN β-1a in the course of disease of the illness is likely to offer the highest chance of potential benefits to prevent further deterioration. Days 1-10 mentioned with each of the investigations noted below refer to the days of IFN β-1a therapy and/or follow-up while in hospital. Patients discharged prior to 10 days from initiation of therapy will, if possible, be seen for medical and study follow-up at 1 month post-initiation of treatment. Every effort will be made to combine clinical care follow-up with study follow-up.

Data available in the medical chart will be collected.

Baseline Investigations:
Given that this is an outbreak with limited health care resources available at the different treatment centres, baseline investigations will be resource-dependent and may include

- Fever/temperature measurement
- PCR determination of viremia
- Clinical evaluation of severity of symptoms such as chills, myalgia, sore throat, cough, nausea, diarrhea, hemorrhage

6.3 Treatment Schedule
IFN β-1a will be obtained from BiogenIdec Inc., Cambridge, MA 02142, USA. The dosage will be as follows: 6 million IU SC × 10 days. Administration should follow the product monograph, and will generally be done by the patient's nurse while in the treatment centre.

The IFN β-1a dose may be decreased if there are problems with tolerance by 3 million IU (15 μg) to 3 million IU. Once it is well tolerated, the dose may remain the same or may be re-increased by 3 million IU to a maximum of 6 million IU/30 μg per day. Dose adjustments are under the treating physician responsibility.

The duration of treatment may be shorter than 10 days in cases where the patient recovers more rapidly, with a minimum of 7 days therapy. The decision to stop IFN β-1a therapy in such cases will be made by the patient's treating physician. In general, patients are expected to discontinue treatment upon discharge from the treatment centre or about 48 hours prior to discharge for observation of deterioration of treatment. Most patients are expected to be admitted at least 7 days due to the nature of the illness. If any patient needs to discontinue prior to day 7 of therapy, the rationale will be documented on a case-by-case basis.

6.4 Concomitant Medication
All concomitant medication should be appropriate as determined by the treating physician and should be documented in the CRF. For instance, at the discretion of the treating physician, broad-spectrum antibiotics may also be given to cover possible superimposed bacterial infections.

6.5 Follow-up
Collection from daily recordings will be undertaken either prospectively or retrospectively to assess adverse event and efficacy of therapy from day 1 (1st day of treatment) to day 10 and then weekly while admitted to the treatment centre and then 30 days post last dose. For patients for whom therapy is discontinued prior to day 10, assessments will follow the same schedule to allow comparison of disease course.
The following will be recorded, as permitted, starting immediately prior to the onset of administration of IFN β-1a, then on a daily basis whilst the patient is in the treatment centre:

- Temperature; fever will be defined as a temperature ≥38 °C.
- Concurrent illness symptoms: chills, myalgia, cough, sore throat, nausea, vomiting, diarrhea, hemaorrhage.
- Adverse events will be recorded per protocol. Medication history at baseline and daily will be recorded.
- Blood samples:
  - 20 cc of Blood for virologic measurement (if feasible).
  - A convalescent serum (10 cc tiger top tube) will be obtained at 1 month (when possible).
- Survival

Of note, it is expected that there will be missing data points for study participants depending on the clinical care data available in the medical chart and residual blood samples available for testing.

7. Clinical Pharmacology of IFN β-1a
7.1 Pharmacodynamics

Interferons (IFNs) are a family of naturally occurring proteins, produced by eukaryotic cells in response to viral infection and other biologic agents. Three major types of interferons have been defined: type I (IFN-alpha, beta, epsilon, kappa and omega), type II (IFN-gamma) and type III (IFN-lambda). Interferon-beta is a member of the type I subset of interferons. The type I interferons have considerably overlapping but also distinct biologic activities. The bioactivities of all IFNs, including IFN-beta, are induced via their binding to specific receptors on the surface of human cells. Differences in the bioactivites induced by the three major subtypes of IFNs likely reflect differences in the signal transduction pathways induced by signaling through their cognate receptors.

Interferon beta exerts its biological effects by binding to the IFNAR1 and IFNAR2 receptor chians on the surface of human cells. This binding initiates a complex cascade of intracellular events that leads to the expression of numerous interferon-induced gene products and markers. These include 2', 5'-oligoadenylate synthetase, β_2-microglobulin, and neopterin. These products have been measured in the serum and cellular fractions of blood collected from patients treated with IFN β-1a.

Clinical studies conducted in multiple sclerosis patients showed that interleukin 10 (IL-10) levels in cerebrospinal fluid were increased in patients treated with IFN β-1a compared to placebo. Serum IL-10 levels maximally were increased by 48 hours after intramuscular injection of IFN β-1a and remained elevated for 1 week. However, no relationship has been established between absolute levels of IL-10 and clinical outcome in multiple sclerosis.

7.2 Pharmacokinetics

Pharmacokinetics of AVONEX in multiple sclerosis patients has not been evaluated. The pharmacokinetic and pharmacodynamic profiles of IFN β-1a in healthy subjects following doses of 30 mcg through 75 micrograms have been investigated. Serum levels of IFN β-1a as measured by antiviral activity are slightly above detectable limits following a 30 mcg subcutaneous dose, and increase with higher doses.

After a subcutaneous dose, serum levels of IFN β-1a generally peak at 15 hours post-dose (range: 6-36 hours) and then decline at a rate consistent with a 19 (range: 8-54) hour elimination half-life.

Biological response markers (e.g., neopterin and β_2-microglobulin) are induced by IFN β-1a following parenteral doses of 15 micrograms through 75 micrograms in healthy subjects and treated patients. Biological response marker levels increase within 12 hours of dosing and remain elevated for at least 4 days. Peak biological response marker levels are typically observed 48 hours after dosing. The relationship of serum IFN β-1a levels or levels of these induced biological response markers to the mechanisms by which IFN β-1a exerts its effects in multiple sclerosis is unknown.

7.3 Description of Pharmaceutical

IFN β-1a Single-Use Prefilled Syringe

A prefilled syringe of IFN β-1a is a sterile liquid for injection. Each 0.5 mL of 60 mcg/mL of IFN β-1a in a prefilled glass syringe contains 30 mcg of IFN β-1a, 0.79 mg Sodium Acetate Trihydrate, USP; 0.25 mg Glacial Acetic Acid, USP; 15.8 mg Arginine Hydrochloride, USP; and 0.025 mg Polysorbate 20 in Water for Injection, USP at a pH of approximately 4.8.

A dose titration device will be supplied by Biogen Idec that will enable a half dose (0.25 mL = 15 μg = 3 million IU) to be administered from the full dose syringe if dose reduction is required.

8. Management of Ebola Disease Progression, Adverse Events and Serious Adverse Events

8.1 Ebola Disease Progression

It is expected that the disease will continue to progress for the initial 24-72 hours of therapy. Stabilization or improvement is not anticipated to manifest until day 3-5. Considering the potential adverse events of IFN β-1a therapy, special consideration will be placed to determine whether the treatment regimen adversely affects disease progression.

If the participant progresses at ≥48 hours while being treated with IFN β-1a in terms of worsening of disease symptoms and if this progression is felt by the treating physician to probably be related to the treatment regimen, then IFN β-1a will be either decreased in dose or discontinued.

8.2 Adverse Events Management

It is expected that IFN β-1a will have a direct antiviral effect if initiated early in the course of illness and will have an immunomodulatory effect on exaggerated inflammatory responses observed in ill patients. It is likely that this drug will cause certain flu-like symptoms or symptoms compatible with disease progression but it is unlikely that these symptoms will be serious with the short 10-day-course. If there is concern that IFN β-1a is associated with worsening of disease, it t may be dose-reduced or treatment stopped.

Adverse Events

This section is extracted from the product monograph and is supplementary information to that provided in section 2.3 of the protocol. The side effects listed are based on a phase 3 study with 9 or 15 μg of IFN β-1a given three times a week for 24-48 weeks. Since IFN β-1a is given for 10 days in this study, the safety information, listed in section 2.3 for 10 days of therapy, may be more relevant.

The most common adverse events of AVONEX reported in placebo-controlled studies (IM administration) are listed below with the incidence in placebo-treated multiple sclerosis patients listed first and AVONEX-treated multiple sclerosis patients listed second: headache (55%, 58%), flu-like symptoms (29%, 49%), pain (21%, 23%), asthenia (18%, 24%), fever (9%, 20%), chills (5%, 19%), abdominal pain (6%, 8%), injection site pain (6%, 8%), infection (4%, 7%), injection site inflammation (2%, 6%), chest pain (2%, 5%), injection site reaction (1%, 3%), toothache

(1%, 3%), depression (14%, 18%), dizziness (12%, 14%), upper respiratory tract infection (12%, 14%), sinusitis (12%, 14%), bronchitis (5%, 8%), nausea (19%, 23%), myalgia (22%, 29%), arthralgia (6%, 9%), urinary tract infection (15%, 17%), urine constituents abnormal (0%, 3%), alopecia (2%, 4%), eye disorder (2%, 4%), injection site ecchymosis (4%, 6%), anemia (1%, 4%), migraine (3%, 5%), vasodilation (0%, 2%). As some of these symptoms may occur following virus infection, it may be difficult to know whether they are due to the administration of IFN β-1a or the patient's underlying condition

Rare Adverse Events (<1% frequency)
Severe psychiatric adverse events may develop in persons receiving therapy with IFN β-1a and includes suicidal ideation and suicide. The risk of this happening is small and less than 1%.
IFN-α/ß has on occasion exacerbated pre-existing cardiovascular disease, and has caused angina (chest pain), rhythm disturbances or heart attacks (myocardial infarction) in these persons. In those with known pre-existing heart disease, physicians will monitor for cardiac symptoms closely and discontinue IFN β-1a if cardiovascular symptoms appear to be worsened by the drug.
IFN β-1a has rarely been associated with ophthalmic problems, including retinal hemorrhages, optic neuritis and papilledema in persons on long-term treatment. Participants will be asked to report to their physician any disturbance in vision while they are on treatment.
IFN β-1a may exacerbate liver disease and will be discontinued with grade 4 hepatotoxicity.
Very rarely IFN β-1a has been associated with pulmonary syndromes including pneumonia and worsening of asthma. If a participant develops worsening pulmonary symptomatology, which is felt to be due to IFN β-1a then the drug will be dose-reduced or stopped.
IFN β-1a may aggravate autoimmune diseases (for example, systemic lupus or rheumatoid arthritis), and those with pre-existing autoimmune disease will be closely monitored.

Management of Adverse Events
Management of adverse events is generally achieved by dose reduction of IFN β-1a, however, in the case of life-threatening adverse events, development of cardiac dysfunction, or precipitous decline in pulmonary function over a 24 hour period, which is not explained by disease progression and which may be associated IFN β-1a, therapy will be discontinued.

Flu-like symptoms can be treated with acetaminophen 500-1000 mg or ibuprofen 200-400 mg 30 minutes before IFN β-1a injection.

The following guidelines will be used to determine when dose modification of the investigational product for an adverse event should be implemented.

- Grade I (mild) adverse events: No dosage adjustment
- Grade 2 (moderate) adverse events: No dosage adjustment
- Grade 3 (severe) adverse events: Patients who develop grade 3 adverse events (except flu like symptoms, unless they are incapacitating) should have IFN β-1a reduced to 3 MIU (15 mcg). Alternatively, IFN β-1a may be discontinued.

- Grade 4 (Life threatening) adverse events (SAE): IFN β-1a will be discontinued.

8.3 Adverse Event Definitions and Reporting

Adverse Event (AE): is any untoward medical occurrence in a patient or clinical investigation participant administered a product. It does not necessarily have to have a causal relationship with this treatment. An AE can therefore be any unfavorable and unintended sign (including an abnormal laboratory finding, for example), symptom or disease temporally associated with the use of a medicinal product, whether or not considered related to the medicinal product.

All adverse events will be recorded and transcribed into the CRF. Solicited AEs and non-solicited AEs will be collected daily for the first four days and then every 2-3 days during therapy, or whenever reported by patients. SAEs will be collected while on study medication and for 30 days after administration of the last dose.

Serious Adverse Event (SAE): is any adverse event that:

-is fatal
-is life-threatening
-results in persistent or significant disability/incapacity
-requires initial hospitalization or prolongation of existing hospitalization
-causes congenital anomalies or birth defects

The term "life-threatening" refers to an event in which the patient was at risk of death at the time of the event. It does not refer to an event, which hypothetically might have caused death, if it were more severe. All deaths occurring while on protocol therapy or within 30 days of the last dose, should be reported (see below).

SAE Reporting

All SAEs must be reported within 24 hours to the Study Coordinator and to enable reporting to the Guinean Ministry of Health within 7 calendar days of identification. All SAEs must also be reported within 24 hours to BiogenIdec Inc. at clinicaltrialsteam@biogenidec.com or by FAX at +1 866 706 5058.

All AEs and SAEs will be evaluated by the investigator or delegate for causal relationship to the investigational product.

8.4 Evaluation of Safety

Safety will be assessed by comparing the occurrence of SAEs and of clinically important adverse events (moderate and severe) attributable to the intervention. Safety monitoring for this interventional study will involve collection of data with direct comparison between IFN β-1a-treated patients and those not receiving IFN β-1a.

Study stopping rules:

- A patient dies during therapy as a result of an SAE assessed as related to the investigational product.

9. Study Endpoints

9.1 Primary

Virologic clearance: Clearance and/or reduction in viral RNA from day 1 to day 10, as determined by PCR and/or quantitative real time PCR. Thus, the presence of virus from any sample will be scored as positive on that day and the number of copies will be compared to evaluate virus clearance with each therapy. Time until virus becomes undetectable.

9.2 Secondary

Safety: Occurrence and severity of adverse event; time to onset, duration (number of days), severity, and seriousness of solicited and non-solicited injection site reactions and systemic adverse events occurring up to 7 days after the last injection. Occurrence, nature, time to onset relative to the first IFN β-1a dose, duration, severity, relationship to injection of SAEs throughout the trial.

- The frequency of clinically important (moderate or severe) adverse events will be compared between the groups
- The frequency of serious adverse events will be compared between the groups.

Parameters to look for adverse events will be solicited such as local injection site erythema and swelling, fever, heart rate, blood pressure, and rash.

9.3 Tertiary

Clinical: Clinical improvement: will be evaluated by comparing the following parameters between the groups:

- Incidence of fever (>38˚C), persistence, and duration.
- Incidence and duration of other symptoms of illness: chills, myalgia, sore throat, cough, nausea, vomiting, diarrhea, and hemorrhage.
- Death – all causes for study duration

Monitoring the clinical course consistently as opposed to looking at the effect of IFN β-1a on a case-by-case basis should provide additional information to guide indications for therapy during the epidemic and provide detailed surveillance data during this public health emergency.

10. Biostatistical Considerations

10.1 Sample size estimate

There is no expected sample size for this pilot clinical trial as it is to prove feasibility. This study can include any patient who is suspected or proven to be infected with EBOV and has been admitted to the treatment centre. A sample size of 30-50 has been chosen to assess feasibility of running a clinical trial in the setting with a large number of patients and also the feasilibty of treating a large number of patients. If successful, a larger clinical trial may be feasible.

10.2 Statistical analyses and methods

Demographics, and underlying conditions will be described by means and standard deviation for continuous variables, by median and interquartile range for ordinal or count variables, and by frequencies and percentages for categorical variables. Endpoints will be described and compared between treatment and usual standard of care groups using cross-tabulation tables and Fisher's Exact test for categorical variables, t-test for normally distributed continuous variables and Wilcoxon non-parametric test for non-normally distributed continuous or ordinal variables.

Safety variables such as fever will be considered in three different formats:

1. As ordinal, representing various levels of severity of the symptom. For instance, fever will be described as "none", "mild", "moderate", or "severe".
2. As binary, considering only the presence vs. the absence of the symptom.
3. As binary, considering the most serious expression of the symptom. For instance by grouping "moderate" and "severe" together.

Separate analyses for time periods of 0-3, 4-7, and 8-10 days will be conducted. For each of these time periods the most severe report of the AE for each participant will be counted.

Adverse Events and Serious Adverse Events will be considered in two ways: presence of any event and presence of any event related to the treatment.

Symptoms will be assessed as a repeated measure using GEE modelling.

Survival will be analyzed using a Kaplan-Meier curve and Cox-proportional hard modeling with 2 week and 1 month survival rates determined.

Viral clearance will be analyzed as binary variables for detection (presence) or non-detection (absence) as well as continuous variables where applicable (e.g. quantitative RT-PCR measurements.

11. Data Management

All data management, including the design of the CRFs, design of the database, as well as data entry and assurance of data integrity will be assumed by the Investigators or delegates. The CRFs, if possible, will be appended to a participant's clinical chart for direct documentation by the treating physician(s) and nurses. This plan is to accommodate data collection and transcription in the context of limited resources during this outbreak.

12. Premature Discontinuation of Treatment

Participants may withdraw from the study at any time for any reason without jeopardy to their current or future care. The investigator may also withdraw patients from the study without the participant's permission in the event of intercurrent illness, adverse events, treatment failures, protocol violation, administrative reasons, or any other reasons. The reason for withdrawal should be clearly documented in the CRF.

Specific criteria for premature discontinuation of treatment will also include, progression of disease due to IFN β-1a or grade IV adverse events felt to be due to IFN β-1a. If the reason for removal of a patient from the study is an adverse event or abnormal laboratory result, the

principal specific event or test will be documented completely on the CRF. A participant will be withdrawn if at any time, in the opinion of the investigator, study participation would not be in the best interest of the participant.

Participants may withdraw or be withdrawn from treatment and this will not automatically lead to withdrawal from the rest of the study. These participants would continue to be followed per protocol for safety and disease course, off therapy. Unnecessary withdrawal of patients should be avoided.

Lost to follow-up procedures
In the case of participants who fail to present for a follow-up examination, documented reasonable effort (i.e.; documented phone calls or certified mail) should be undertaken to locate or recall them or at least to determine their health status while fully respecting the participant's rights. These efforts should be documented in the participant's source documents and CRF.

Termination classification
The investigator will classify the termination status of each participant at the end of the study in the termination page of the CRF according to the following:

- Serious Adverse Event
- Other Adverse Event
- Non-compliance with protocol
- Lost to follow-up
- Voluntary withdrawal not for an Adverse Event
- Withdrawal by Investigator with rationale

Follow-up of discontinuation
Participants who withdraw for an adverse event related to IFN β-1a should be followed as deemed appropriate by the investigator until resolution of the event or until chronicity of the event has been established. In cases where the adverse event is not related to IFN β-1a, the participant should be followed as deemed appropriate by the investigator. Documentation is deemed as that in the source documents and as indicated in the CRF. In the event of a loss to follow-up, the loss to follow-up procedures will be followed. As noted above, participants may be withdrawn from investigational product therapy but continue to be followed according to protocol for their clinical course and/or by providing additional blood and respiratory secretion specimens for research testing.

Interruption of the study
The study may be discontinued for administrative reasons or if new data about the investigational product resulting from this or other studies become available, and/or on advice of the regulatory authorities. If the trial is prematurely terminated for any reason, the investigator should inform the study participants in a timely fashion and should assure appropriate therapy and follow-up.

Modifications of the study protocol
No amendments to this study will be made without consultation with, and agreement of, the study sponsors, including the manufacturer of the IFN β-1a i.e. BiogenIdec, Inc. Any

amendment to the study that appears necessary during the course of the study must be discussed by the investigator/physician and the sponsor-investigator concurrently. If there is a need for an amendment, it will become a formal part of the protocol and will undergo appropriate approvals (regulatory authorities). An amendment may be implemented prior to regulatory approval in the event that it is necessary to eliminate apparent immediate hazards to the participants.

An administrative change to the protocol is one that modifies administrative and logistical aspects of a protocol but does not affect the participants' safety, the objectives of the study and its progress. An administrative change can be sent as a notification, as it does not require approval by the Guinean Ministry of Health prior to implementation.

13. Conduct of the Investigation

This study will be conducted in accordance with the Declaration of Helsinki, as well as the International Conference on Harmonization (ICH) Good Clinical Practices (GCP), applicable national and local requirements by the respective authorities (Regulatory Agencies such as Health Protection Branch in Canada; REB/IRB). The investigators will be familiar with the principles elaborated in the Declaration of Helsinki and will adhere to these principles, as well as the GCP guidelines and other local and regional applicable legislations and regulations (e.g. privacy legislation).

14. Ethics Review and Consent

The study and the appropriate documents (protocol, informed consent, product monograph, subject recruitment tools, and any other written information to be provided to the participants) will be reviewed by an REB/IEC of record for each respective treatment centre planning to conduct the study. Before inclusion of the first participant at the site, the study must be approved by, or receive a favorable opinion from, the appropriate REB/IEC. A sample consent form can be found in the application.

Each investigator is responsible for obtaining this approval, or favorable opinion, before the start of the trial and with any subsequent amendment in compliance with Good Clinical Practice (GCP) and local regulations. Copies of these approvals, along with information on the type, version number, and date of the document(s), and the date of approval, must be forwarded to the sponsor-investigator, or delegate. All SAEs occurring during the trial will be reported to the REB/IEC, according to the local REB/IEC policy by the site investigator, as well as to the sponsor (see Safety reporting section of the protocol).

15. Time Frame for the Study

Given the nature of this EBOV outbreak, it is difficult to predict the waves and the study may span more than one wave, and thus it is difficult to determine the actual time to complete the study.

Since this is a pilot study, it is important to have such a protocol in readiness so that, new cases can be immediately enrolled. It is estimated that during a wave, it will be possible to recruit 30 participants, in less than one month. It is planned to propose the study to be conducted at other treatment centres across West Africa. The rationale is to increase the rate of enrolment for the pilot study so that if a potential benefit is detected early, IFN β-1a therapy can be evaluated

relatively rapidly, in an effort to make it available, through the extended study (and possibly a larger multicentre international study) as part of the treatment armamentarium as early as possible.

16. Analysis of Final Data

The initial results of the pilot trial will determine if it should be extended (extension to 100 participants per this protocol) and whether a larger multicenter study will be appropriate. Determining factors will be safety and, if possible with the small sample size, a trend toward efficacy when compared to the standard of care regimen. In addition, the effect of the treatment regimen on viral detection will be examined.

REFERENCES

1. Hutchinson, K.L., Rollin, P.E. (2007) Cytokine and chemokine expression expression in humans infected with Sudan Ebola virus. J. Infect. Dis. 196: S357-363.
2. McElroy, A.K. *et al.* (2014) Ebola hemorrhagic fever: novel biomarker correlates of clinical outcome. J. Infect. Dis. 10: 558-566.
3. Kuhl, A., Pohlmann, S. (2012) How Ebola virus counters the interferon system. Zoonoses Public Health 59: 116-131.
4. Cardenas, W.B. (2010) Evasion of the interferon-mediated antiviral response by filoviruses. Viruses 2: 262-282.
5. Yen, B. *et al.* (2014) Molecular basis for Ebola virus VP35 suppression of humans dendritic cell maturation. J. Virol. Aug. 20 [Epub ahead of print].
6. Smith, L.M. *et al.* (2013) Interferon β therapy prolongs survival in rhesus macaque models of Ebola and Marburg hemorrhagic fever. J. Infect. Dis. 208: 310-318.
7. Jahrling, P.B. *et al.* (1999) Evaluation of immune globulin and recombinant interferon-α2b for treatment of experimental Ebola virus infections. J. Infect. Dis. 179: S224-234.
8. Qiu, X. *et al.* (2013) mABs and Ad-vectored IFN-α therapy rescue Ebola-infected nonhuman primates when administered after the detection of viremia and symptoms. Sci. Transl. Med. 5: 207ra 143.
9. Qiu X. *et al.* (2013) Monoclonal antibodies combined with adenovirus-vectored interferon significantly extend the treatment window in Ebola virus-infected guinea pigs. J. Virol. 87: 7754-7757.
10. Geisbert, T.W. *et al.* (2003) Pathogenesis of Ebola hemorrhagic fever in cynomologous macaques: evidence that dendritic cells are early and sustained targets of infection. Am. J. Pathol. 163: 2347-2370.
11. Schramm, L.M.*et al.* (2012) High-throughput quantitative real-time PCR array for absolute and relative quantification of rhesus macaque types I, II and II interferon and their subtypes. J IFN and cytokine Res. 32: 407-415.
12. Puig, M. *et al.* (2012) TLR9 and TLR7 agonists mediate distinct type I IFN responses in humans and nonhuman primates *in* vitro and *in vivo.*J. Leuk. Biol. 91: 147-158.
13. Jeong, A.R. *et al.* (2008) Gene expression profile of Th1 and Th2 cytokines and their receptors in human and nonhuman primates. J. Med. Primatol. 37: 290-296.
14. Bett, A.J. *et al.* (2010) Comparison of T cell immune responses induced by vectored HIV vaccines in NHPs and humans. Vaccine 84: 9810-9816.

15. Habersetzer, F. *et al.* (2000) A pilot study of recombinant interferon beta-1a for the treatment of chronic hepatitis C. Liver 20:437-441.
16. Festi, D. *et al.* (2004) Safety of interferon β treatment for chronic HCV hepatitis. World J. Gastroenterol. 10:12-16.

17. Arase, Y. et al (2011) Efficacy and safety of combination therapy of natural human interferon beta and ribavirin in chronic hepatitis C patients. Intern. Med. 50:2083-2088.
