# Supplementary material for: Interferon β-1a for the treatment of Ebola virus disease: A historically controlled, single-arm proof-of-concept trial
Source: PLoS One. 2017 Feb 22;12(2):e0169255. doi: 10.1371/journal.pone.0169255 (PMC5321269; doi:10.1371/journal.pone.0169255)
Supplement: S1 Table — (DOCX) [file pone.0169255.s002.docx]

## S1 Table All patient analysis: baseline characteristics and regression analysis

for treatment effects on survival

| **Variable** | **Categories** | **Controls (n=38)** | **IFN β-1a( n=9)** | **p-value** |
| --- | --- | --- | --- | --- |
| Age | Median(Range) | 28(20-70) | 38(18-50) | 0.68 |
| Sex | Female | 25(65.8%) | 5(55.6%) | 0.7 |
|  | Male | 13(34.2%) | 4(44.4%) |  |
| CT | Median(Range) | 19(14-33.6) | 22.1(16.2-30.6) | 0.041 |
| Status | Alive | 6(15.8%) | 6(66.7%) | 0.0048 |
|  | Deceased | 32(84.2%) | 3(33.3%) |  |

| **Variable** | **OR** | **p-value** |
| --- | --- | --- |
| IFN | 0.13 | 0.022 |
| CT value | 0.84 | 0.06 |
