# Supplementary material for: Interferon β-1a for the treatment of Ebola virus disease: A historically controlled, single-arm proof-of-concept trial
Source: PLoS One. 2017 Feb 22;12(2):e0169255. doi: 10.1371/journal.pone.0169255 (PMC5321269; doi:10.1371/journal.pone.0169255)
Supplement: S2 Table — (DOCX) [file pone.0169255.s003.docx]

## S2 Table Subset analysis^*^: baseline characteristics and regression analysis for

treatment effects on survival

| **Variable** | **Categories** | **Controls (n=28)** | **IFN β-1a (n= 9)** | **p-value** |
| --- | --- | --- | --- | --- |
| Age | Median(Range) | 28(20-50) | 38(18-50) | 0.85 |
| Sex | Female | 18(64.3%) | 5(55.6%) | 0.7 |
|  | Male | 10(35.7%) | 4(44.4%) |  |
| CT | Median(Range) | 20(16.2-26.5) | 22.1(16.2-30.6) | 0.086 |
| Status | Alive | 5(17.9%) | 6(66.7%) | 0.011 |
|  | Deceased | 23(82.1%) | 3(33.3%) |  |

| **Variable** | **OR** | **p-value** |
| --- | --- | --- |
| IFN | 0.16 | 0.043 |
| CT value | 0.82 | 0.13 |

^*^ infected and untreated control patients older than 50 years of age and with baseline CT values <16 or >31 were excluded.
