# Supplementary material for: Interferon β-1a for the treatment of Ebola virus disease: A historically controlled, single-arm proof-of-concept trial
Source: PLoS One. 2017 Feb 22;12(2):e0169255. doi: 10.1371/journal.pone.0169255 (PMC5321269; doi:10.1371/journal.pone.0169255)
Supplement: S3 Table — For each patient in the IFN group an attempt was made to match with untreated patients who had a less than 5 years difference in age and less than 2 units difference in their baseline CT value. For 5 patients suitable matches were found: For IFN 02 one match, for IFN 03 one match, for IFN 04 three matches, for IFN 08 two matches and for IFN 10 three matches. For IFN 05, IFN 06, IFN 07 and IFN 09 no matches were found and they did not enter this analysis. (DOCX) [file pone.0169255.s004.docx]

**S3 Table** Matched analysis^*^ : baseline characteristics and regression analysis for treatment effects on survival

| **Variable** | **Categories** | **Controls (n=10)** | **IFN β-1a (n=5)** | **p-value** | **Standardized differences** |
| --- | --- | --- | --- | --- | --- |
| age | Median(Range) | 25.5(20-52) | 21(18-50) | 0.38 | 16.7 |
| sex | Female | 7(70%) | 2(40%) | 0.33 | 63.25 |
|  | Male | 3(30%) | 3(60%) |  | 63.25 |
| CT | Median(Range) | 20.7(16.2-26.5) | 21.2(16.2-26.8) | 1 | 16.69 |
| status | Alive | 1(10%) | 2(40%) | 0.24 | 73.85 |
|  | Deceased | 9(90%) | 3(60%) |  | 73.85 |

^*^ For each patient in the IFN group an attempt was made to match with untreated patients who had a less than 5 years difference in age and less than 2 units difference in their baseline CT value. For 5 patients suitable matches were found: For IFN 02 one match , for IFN 03 one match, for IFN 04 three matches, for IFN 08 two matches and for IFN 10 three matches. For IFN 05, IFN 06, IFN 07 and IFN 09 no matches were found and they did not enter this analysis.
