# Supplementary material for: Interferon β-1a for the treatment of Ebola virus disease: A historically controlled, single-arm proof-of-concept trial
Source: PLoS One. 2017 Feb 22;12(2):e0169255. doi: 10.1371/journal.pone.0169255 (PMC5321269; doi:10.1371/journal.pone.0169255)
Supplement: S4 Table — Symptoms recorded am, pm and e (evening), as indicated. With reference to asthenia pm (highlighted in grey), this symptom remained relatively unchanged over the 3 time periods examined among the historic controls receiving supporting care only (54%, 74%, 56%), and the p-value is not significant (p = 0.47). For the IFN ß-1a treated patients, the incidence of asthenia (pm) decreases over the 3 time periods (69%, 43%, 8%) with the OR in this arm of 0.65 and p<0.0001. Comparing the OR between historic controls and IFN-treated, the OR in the control arm is 1.65 fold larger than the OR in the IFN-treated arm, hence the significance recorded in the last column in Table 3. (DOCX) [file pone.0169255.s005.docx]

**S4 Table** Effects of IFN β-1a on frequency of clinical symptoms associated with EVD

| **Symptom** | **Days** | **Controls**  **assessed** | **Controls with symptom** | **IFN β-1a treated**  **assessed** | **IFN β-1a treated with symptom** |
| --- | --- | --- | --- | --- | --- |
| headache_am | [0-2] | 59 | 7(12%) | 27 | 5(19%) |
| headache_am | [3-5] | 43 | 4(9%) | 23 | 2(9%) |
| headache_am | [6-9] | 25 | 9(36%) | 24 | 2(8%) |
| headache_pm | [0-2] | 59 | 16(27%) | 26 | 4(15%) |
| headache_pm | [3-5] | 43 | 5(12%) | 23 | 2(9%) |
| headache_pm | [6-9] | 25 | 3(12%) | 24 | 2(8%) |
| headache_e | [0-2] | 59 | 11(19%) | 26 | 4(15%) |
| headache_e | [3-5] | 43 | 4(9%) | 23 | 3(13%) |
| headache_e | [6-9] | 25 | 5(20%) | 23 | 2(9%) |
| asthenia_am | [0-2] | 59 | 31(53%) | 27 | 17(63%) |
| asthenia_am | [3-5] | 43 | 32(74%) | 23 | 11(48%) |
| asthenia_am | [6-9] | 25 | 15(60%) | 24 | 2(8%) |
| asthenia_pm | [0-2] | 59 | 32(54%) | 26 | 18(69%) |
| asthenia_pm | [3-5] | 43 | 32(74%) | 23 | 10(43%) |
| asthenia_pm | [6-9] | 25 | 14(56%) | 24 | 2(8%) |
| asthenia_e | [0-2] | 59 | 31(53%) | 26 | 14(54%) |
| asthenia_e | [3-5] | 43 | 25(58%) | 23 | 9(39%) |
| asthenia_e | [6-9] | 25 | 12(48%) | 23 | 1(4%) |
| muscle_pain_am | [0-2] | 59 | 14(24%) | 27 | 6(22%) |
| muscle_pain_am | [3-5] | 43 | 13(30%) | 23 | 5(22%) |
| muscle_pain_am | [6-9] | 25 | 5(20%) | 24 | 4(17%) |
| muscle_pain_pm | [0-2] | 59 | 14(24%) | 26 | 5(19%) |
| muscle_pain_pm | [3-5] | 43 | 15(35%) | 23 | 8(35%) |
| muscle_pain_pm | [6-9] | 25 | 4(16%) | 24 | 3(12%) |
| muscle_pain_e | [0-2] | 59 | 18(31%) | 26 | 3(12%) |
| muscle_pain_e | [3-5] | 43 | 8(19%) | 23 | 3(13%) |
| muscle_pain_e | [6-9] | 25 | 6(24%) | 23 | 2(9%) |
| anorexia_am | [0-2] | 59 | 18(31%) | 27 | 5(19%) |
| anorexia_am | [3-5] | 43 | 15(35%) | 23 | 3(13%) |
| anorexia_am | [6-9] | 25 | 11(44%) | 24 | 0(0%) |
| anorexia_pm | [0-2] | 59 | 13(22%) | 26 | 4(15%) |
| anorexia_pm | [3-5] | 43 | 17(40%) | 23 | 3(13%) |
| anorexia_pm | [6-9] | 25 | 11(44%) | 24 | 1(4%) |
| anorexia_e | [0-2] | 59 | 13(22%) | 26 | 2(8%) |
| anorexia_e | [3-5] | 43 | 10(23%) | 23 | 2(9%) |
| anorexia_e | [6-9] | 25 | 6(24%) | 23 | 0(0%) |
| nausea_am | [0-2] | 59 | 5(8%) | 27 | 1(4%) |
| nausea_am | [3-5] | 43 | 2(5%) | 23 | 1(4%) |
| nausea_am | [6-9] | 25 | 2(8%) | 24 | 0(0%) |
| nausea_pm | [0-2] | 59 | 10(17%) | 26 | 1(4%) |
| nausea_pm | [3-5] | 43 | 4(9%) | 23 | 0(0%) |
| nausea_pm | [6-9] | 25 | 0(0%) | 24 | 0(0%) |
| vomiting_am | [0-2] | 59 | 8(14%) | 27 | 12(44%) |
| vomiting_am | [3-5] | 43 | 13(30%) | 23 | 6(26%) |
| vomiting_am | [6-9] | 25 | 3(12%) | 24 | 0(0%) |
| vomiting_pm | [0-2] | 59 | 20(34%) | 26 | 13(50%) |
| vomiting_pm | [3-5] | 43 | 6(14%) | 23 | 4(17%) |
| vomiting_pm | [6-9] | 25 | 3(12%) | 24 | 0(0%) |
| vomiting_e | [0-2] | 59 | 13(22%) | 26 | 6(23%) |
| vomiting_e | [3-5] | 43 | 10(23%) | 23 | 4(17%) |
| vomiting_e | [6-9] | 25 | 2(8%) | 23 | 1(4%) |
| diarrhea_am | [0-2] | 59 | 15(25%) | 27 | 20(74%) |
| diarrhea_am | [3-5] | 43 | 24(56%) | 23 | 11(48%) |
| diarrhea_am | [6-9] | 25 | 11(44%) | 24 | 2(8%) |
| diarrhea_pm | [0-2] | 59 | 26(44%) | 26 | 16(62%) |
| diarrhea_pm | [3-5] | 43 | 19(44%) | 23 | 8(35%) |
| diarrhea_pm | [6-9] | 25 | 10(40%) | 24 | 0(0%) |
| diarrhea_e | [0-2] | 59 | 22(37%) | 26 | 14(54%) |
| diarrhea_e | [3-5] | 43 | 20(47%) | 23 | 7(30%) |
| diarrhea_e | [6-9] | 25 | 11(44%) | 23 | 0(0%) |
| dyspnea_pm | [0-2] | 59 | 4(7%) | 26 | 0(0%) |
| dyspnea_pm | [3-5] | 43 | 0(0%) | 23 | 2(9%) |
| dyspnea_pm | [6-9] | 25 | 3(12%) | 24 | 2(8%) |
| dyspnea_e | [0-2] | 59 | 4(7%) | 26 | 0(0%) |
| dyspnea_e | [3-5] | 43 | 3(7%) | 23 | 1(4%) |
| dyspnea_e | [6-9] | 25 | 2(8%) | 23 | 1(4%) |
| cough_am | [0-2] | 59 | 5(8%) | 27 | 7(26%) |
| cough_am | [3-5] | 43 | 3(7%) | 23 | 7(30%) |
| cough_am | [6-9] | 25 | 7(28%) | 24 | 3(12%) |
| cough_pm | [0-2] | 59 | 3(5%) | 26 | 7(27%) |
| cough_pm | [3-5] | 43 | 1(2%) | 23 | 5(22%) |
| cough_pm | [6-9] | 25 | 3(12%) | 24 | 3(12%) |
| cough_e | [0-2] | 59 | 3(5%) | 26 | 4(15%) |
| cough_e | [3-5] | 43 | 3(7%) | 23 | 4(17%) |
| cough_e | [6-9] | 25 | 5(20%) | 23 | 2(9%) |
| thor_pain_am | [0-2] | 59 | 1(2%) | 27 | 6(22%) |
| thor_pain_am | [3-5] | 43 | 0(0%) | 23 | 3(13%) |
| thor_pain_am | [6-9] | 25 | 0(0%) | 24 | 2(8%) |
| thor_pain_pm | [0-2] | 59 | 3(5%) | 26 | 4(15%) |
| thor_pain_pm | [3-5] | 43 | 0(0%) | 23 | 1(4%) |
| thor_pain_pm | [6-9] | 25 | 0(0%) | 24 | 2(8%) |
| abd_pain_am | [0-2] | 59 | 7(12%) | 27 | 16(59%) |
| abd_pain_am | [3-5] | 43 | 15(35%) | 23 | 6(26%) |
| abd_pain_am | [6-9] | 25 | 4(16%) | 24 | 0(0%) |
| abd_pain_pm | [0-2] | 59 | 18(31%) | 26 | 8(31%) |
| abd_pain_pm | [3-5] | 43 | 15(35%) | 23 | 3(13%) |
| abd_pain_pm | [6-9] | 25 | 1(4%) | 24 | 0(0%) |
| abd_pain_e | [0-2] | 59 | 10(17%) | 26 | 6(23%) |
| abd_pain_e | [3-5] | 43 | 15(35%) | 23 | 1(4%) |
| abd_pain_e | [6-9] | 25 | 2(8%) | 23 | 0(0%) |
| dehydration_e | [0-2] | 59 | 4(7%) | 26 | 1(4%) |
| dehydration_e | [3-5] | 43 | 5(12%) | 23 | 0(0%) |
| dehydration_e | [6-9] | 25 | 2(8%) | 23 | 0(0%) |
| hemorrage_am | [0-2] | 59 | 2(3%) | 27 | 1(4%) |
| hemorrage_am | [3-5] | 43 | 5(12%) | 24 | 6(25%) |
| hemorrage_am | [6-9] | 25 | 2(8%) | 29 | 7(24%) |
| hemorrage_pm | [0-2] | 59 | 4(7%) | 27 | 1(4%) |
| hemorrage_pm | [3-5] | 43 | 5(12%) | 24 | 7(29%) |
| hemorrage_pm | [6-9] | 25 | 1(4%) | 29 | 8(28%) |
| hemorrage_e | [0-2] | 59 | 5(8%) | 27 | 0(0%) |
| hemorrage_e | [3-5] | 43 | 1(2%) | 24 | 7(29%) |
| hemorrage_e | [6-9] | 25 | 3(12%) | 29 | 6(21%) |
| epigastralgia_am | [0-2] | 59 | 5(8%) | 27 | 7(26%) |
| epigastralgia_am | [3-5] | 43 | 2(5%) | 24 | 5(21%) |
| epigastralgia_am | [6-9] | 25 | 1(4%) | 29 | 1(3%) |
| epigastralgia_pm | [0-2] | 59 | 5(8%) | 27 | 7(26%) |
| epigastralgia_pm | [3-5] | 43 | 4(9%) | 24 | 4(17%) |
| epigastralgia_pm | [6-9] | 25 | 2(8%) | 29 | 1(3%) |
| epigastralgia_e | [0-2] | 59 | 9(15%) | 27 | 5(19%) |
| epigastralgia_e | [3-5] | 43 | 2(5%) | 24 | 2(8%) |
| epigastralgia_e | [6-9] | 25 | 1(4%) | 29 | 1(3%) |
| arthralgia_am | [0-2] | 59 | 12(20%) | 27 | 6(22%) |
| arthralgia_am | [3-5] | 43 | 8(19%) | 23 | 5(22%) |
| arthralgia_am | [6-9] | 25 | 4(16%) | 25 | 5(20%) |
| arthralgia_pm | [0-2] | 59 | 13(22%) | 26 | 4(15%) |
| arthralgia_pm | [3-5] | 43 | 7(16%) | 23 | 8(35%) |
| arthralgia_pm | [6-9] | 25 | 3(12%) | 25 | 4(16%) |
| arthralgia_e | [0-2] | 59 | 11(19%) | 26 | 3(12%) |
| arthralgia_e | [3-5] | 43 | 6(14%) | 23 | 3(13%) |
| arthralgia_e | [6-9] | 25 | 6(24%) | 23 | 2(9%) |

thor ~ thoracic; abd ~ abdominal.

Symptoms recorded am, pm and e (evening), as indicated.

With reference to asthenia pm (highlighted in grey), this symptom remained relatively unchanged over the 3 time periods examined among the historic controls receiving supporting care only (54%, 74%, 56%), and the p-value is not significant (p=0.47). For the IFN ß-1a treated patients, the incidence of asthenia (pm) decreases over the 3 time periods (69%, 43%, 8%) with the OR in this arm of 0.65 and p<0.0001. Comparing the OR between historic controls and IFN-treated, the OR in the control arm is 1.65 fold larger than the OR in the IFN-treated arm, hence the significance recorded in the last column in Table 3.
